# Ancient genes establish stress-induced mutation as a hallmark of cancer

**DOI:** 10.1371/journal.pone.0176258

**Published:** 2017-04-25

**Authors:** Luis Cisneros, Kimberly J. Bussey, Adam J. Orr, Milica Miočević, Charles H. Lineweaver, Paul Davies

**Affiliations:** 1 NantOmics, Tempe, Arizona, United States of America; 2 BEYOND Center for Fundamental Concepts in Science, Arizona State University, Tempe, Arizona, United States of America; 3 Department of Biomedical Informatics, Arizona State University, Tempe, Arizona, United States of America; 4 School of Life Sciences, Arizona State University, Tempe, Arizona, United States of America; 5 Department of Psychology, Arizona State University, Tempe, Arizona, United States of America; 6 Planetary Science Institute, Research School of Astronomy and Astrophysics and Research School of Earth Sciences, Australian National University, Canberra, Australian Capital Territory, Australia; CNR, ITALY

## Abstract

Cancer is sometimes depicted as a reversion to single cell behavior in cells adapted to live in a multicellular assembly. If this is the case, one would expect that mutation in cancer disrupts functional mechanisms that suppress cell-level traits detrimental to multicellularity. Such mechanisms should have evolved with or after the emergence of multicellularity. This leads to two related, but distinct hypotheses: 1) Somatic mutations in cancer will occur in genes that are younger than the emergence of multicellularity (1000 million years [MY]); and 2) genes that are frequently mutated in cancer and whose mutations are functionally important for the emergence of the cancer phenotype evolved within the past 1000 million years, and thus would exhibit an age distribution that is skewed to younger genes. In order to investigate these hypotheses we estimated the evolutionary ages of all human genes and then studied the probability of mutation and their biological function in relation to their age and genomic location for both normal germline and cancer contexts. We observed that under a model of uniform random mutation across the genome, controlled for gene size, genes less than 500 MY were more frequently mutated in both cases. Paradoxically, causal genes, defined in the COSMIC Cancer Gene Census, were depleted in this age group. When we used functional enrichment analysis to explain this unexpected result we discovered that COSMIC genes with recessive disease phenotypes were enriched for DNA repair and cell cycle control. The non-mutated genes in these pathways are orthologous to those underlying stress-induced mutation in bacteria, which results in the clustering of single nucleotide variations. COSMIC genes were less common in regions where the probability of observing mutational clusters is high, although they are approximately 2-fold more likely to harbor mutational clusters compared to other human genes. Our results suggest this ancient mutational response to stress that evolved among prokaryotes was co-opted to maintain diversity in the germline and immune system, while the original phenotype is restored in cancer. Reversion to a stress-induced mutational response is a hallmark of cancer that allows for effectively searching “protected” genome space where genes causally implicated in cancer are located and underlies the high adaptive potential and concomitant therapeutic resistance that is characteristic of cancer.

## Introduction

A defining quality of life is its phenotypic plasticity, generated through the ability to regulate gene expression and other cellular functions in response to environmental factors, critical properties that enable organisms to respond to a wide variety of environmental challenges in a coordinated and systematic way [1–4]. Yet when confronted with persistent unfavorable conditions, primitive life forms could exhibit more dramatic and evolutionarily deep-rooted responses. In these circumstances, a population of microorganisms is likely to face extinction unless an appropriate adaptation is promptly deployed. A prime example of an adaptive strategy is for cells to elevate their rate of genetic mutation in order to increase the probability of discovering a solution to their burden. Mechanisms such as slipped-strand mispairing, polymerase slippage, gene amplification, deregulation of mismatch repair, and recombination between imprecise homologies underlie the generation of genetic alterations at high frequencies under specific conditions [5–7]. These genotypic alterations can promote phenotypic heterogeneity and adaptive potential in clonal populations of cells, even during stationary growth phases, a process that has been termed “adaptive mutation” [7–14]. Such mechanisms allow for heightened exploration of the phenotypic landscape during conditions of stress, leading to higher rates of effective evolution; a condition well illustrated with the popular proverb: “necessity is the mother of invention”.

Cancer is a disease of bodies, and therefore of multicellular organisms, yet many of the hallmarks of cancer [15,16] suggest an atavistic reversion to an ancestral single-celled phenotype. For cancer cells, the body is no longer a larger functioning organism to which they belong and support, but a complex host ecosystem that they adapt to in order to survive and thrive. From a theoretical standpoint, the emergence of multicellularity represents an increase in the complexity of life in which cells became cooperative aggregates because of the balance between cellular conflict and collective fitness. This transition requires the evolution of both cooperation-promoting and conflict-reducing adaptations [17]. While the mechanisms for adaptive mutation are essential for the survival of single celled organisms exposed to stress, somatic cells in multicellular organisms typically reside in stable homeostatic conditions and are thus “protected” from the drastic changes in the environment that demand engaging in such heritable responses. Furthermore, the integrity of the multicellular structure demands global genetic coherence and strong inhibition of independent somatic cell evolution, although phenotypic plasticity, sometimes heritable, is required in order to maintain function in the face of organism level stresses that place large, differential demands on organs [18,19]. It is well known that genes associated with cancer have phylogenetic origins associated with the emergence of multicellularity [20–23]. For example, the genomic analysis by Domazet-Lošo and Tautz [21] based on four different cancer gene datasets demonstrated that the origin of gatekeeper oncogenes coincides with a pronounced phylostratigraphic peak at the onset of Metazoa. It therefore seems plausible that a source of stress capable of breaking the homeostatic equilibrium in the milieu could trigger an ancestral adaptive mutation program in a somatic cell, inducing genetic instability that could result in cancer if not suppressed by other mechanisms. In this context cancer can be understood as a relaxation of the genetic constraints evolved to maintain the complex structure of multi-cellularity, resulting in a relaxation of constraints that suppress individual somatic cell evolution[20–24].

Here we present evidence demonstrating that cancer manifests as an atavistic recapitulation of pre-metazoan [24] mechanisms of stress-induced mutation in somatic cells, explaining its capacity to evolve resistance to therapy. The mechanistic roots of this behavior are retained over evolutionary time scales because they are critical to the successful function of the germline and immune system. In addition to generating base-line diversity in both the innate and adaptive immune system, normal germline mutational patterns maintain diversity in recently evolved gene families governing functions such as toxin detection and detoxification. In cancer the controlled restriction of this phenomenon to the germline and immune system is disrupted, allowing somatic cells to effectively search ancient genome space for solutions to the stress-induced pressures they are experiencing. We propose stressed-induced mutation as a hallmark of cancer reflected by genomic instability.

## Methods

### Gene ageing

Gene homologies represent the evolutionary history of gene families. Accordingly, an ortholog of a human gene found in any other species can be assumed to have diverged from a common ancestor. Thus, by grouping orthologous genes into gene families, the age of the human gene can be identified by the divergence time of the last common ancestor of all the species contained within the gene family.

Given that this approach is contingent on the definition of homology, more accurate gene family builds will lead to better estimations of gene ages. We looked at three pertinent homology databases to identify the one with the most coverage across all kingdoms of life and the most robust human gene families. We considered Ensembl Compara / Ensembl Pan-Taxonomic Compara [25,26] (release 22, containing 19,756 genes), NCBI HomoloGene [27] (18,304 genes) and HOGENOM [28] (17,086 genes from the nucleotide database). We chose these databases because they cover many species across all taxonomic groups and use both protein and genetic sequence comparisons with sophisticated phylogenetic reconciliation methods to predict evolutionary trees across the whole set of protein-coding genes and non-coding RNA (ncRNA) genes [26]. Based on our analysis (see S1 Text for details) we selected the Ensembl Compara/Ensembl Pan-Taxonomic Compara database as the best option for generating gene families in our ageing method.

We then determined gene ages as the maximum phylogenetic divergence time between humans and all the species represented in each corresponding gene family according to the TimeTree database [29]. The age of a common ancestor in an evolutionary tree is always older than the divergence time between all the species branching out from it, therefore this measure provides an estimate of the minimum expected age of the gene.

### Cancer gene list

Sanger’s Catalogue Of Somatic Mutations In Cancer (COSMIC) is a comprehensive resource of somatic mutations in human cancer [30,31]. We studied 458 genes with mutations that have been causally implicated in cancer (Cancer Gene Census), and categorized each as dominant, recessive, or both according to COSMIC annotations. This classification is based on whether a single allele (dominant) or multiple alleles (recessive) must be mutated in order to observe a cancer phenotype.

### Genomic analysis

We obtained variants called from whole genome sequence (WGS) samples from the International Cancer Genomics Consortium (ICGC) data portal [32] (release 19) for a total of 764 samples from a variety of tissues, including: pancreas (262), prostate (198), ovarian (115), bone (97), skin (59), blood (26), brain (4) and 3 samples with unknown tissue of origin (see Table C in S1 Text for a list of references per project and S4 Table for a list of specific donors and samples). We also obtained normal tissue variants data from the Complete Genomics Indices database in the 1000 Genome Project [33] (release 20130502, see S4 Table for list of donors). In this case, we mined 129 WGS trio samples to identify private variations (i.e. present in the donor but not in either parent). We parsed this data to identify the genomic locations of both double strand break (DSB) events comprising complex multi-base variations, section deletions/insertions and other DNA rearrangements and single nucleotide variants (SNV) representing deletions, insertions, and substitutions of one or two bases. Alterations in regions containing single-, di-, and tri-nucleotide repeats where strand slippage could account for larger rearrangements were also classified as SNVs.

A priori, we removed all events occurring in regions known to be involved in somatic hypermutation [34,35] to avoid biasing the clustering analysis. A group of SNVs were determined to be in a cluster if the distance between two SNVs was less than 25 kb, the cluster had at least 3 SNVs, and the probability of finding such a grouping of SNVs by chance was less than 1% [35]. Hotspotting of clusters was determined by evaluating the number cluster centers observed inside intervals of 1 kb across each chromosome. The expected value of events in the interval was given by the total number of events divided by the number of intervals in the chromosome. Using a binomial test we determined if the observed number of events was larger, smaller, or close to the statistically expected value based on a uniform distribution of events, defining the interval as a hot, cold, or null region, respectively. Odds ratios were computed using a Fisher’s Exact test as implemented in the fisher.test function in R.

### Functional enrichment analysis

We used the Functional Enrichment clustering tool of DAVID [36,37] to determine cellular functions over-represented in various gene lists. This tool evaluates gene sets for enrichment across multiple ontologies and then groups the resulting enriched functions into clusters defined by maximizing the overlap of gene membership within the enriched functions. We designated genes by their Ensembl ID and enrichment using all human genes as the background list for comparison unless otherwise specified. The default ontologies and stringency settings were used for all analyses. We reported functional enrichment if the Benjamini-Hockenburg [38] corrected p-value was less than 0.05 for 3 or more categories within a cluster.

## Results

### Mutational frequency of human genes as a function of evolutionary age

The atavistic model of cancer presumes that the cancer phenotype is to some degree an evolutionarily conserved ‘genetic subroutine’ that is suppressed by multicellularity but becomes re-activated through oncogenic progression [24,39]. There are two related, but distinct hypotheses that result from this model: 1) Somatic mutation will avoid regions of the genome with deep-evolutionary roots, and thus occur in genes that are younger than the emergence of multicellularity (1000 million years [MY]); and 2) genes that are frequently mutated in cancer and whose mutations trigger the cancer subroutine evolved between 500–1000 MYA or with the evolution of complex multicellularity (<500 MYA), and thus would exhibit an age distribution that is skewed to younger genes.

**Fig 1:**
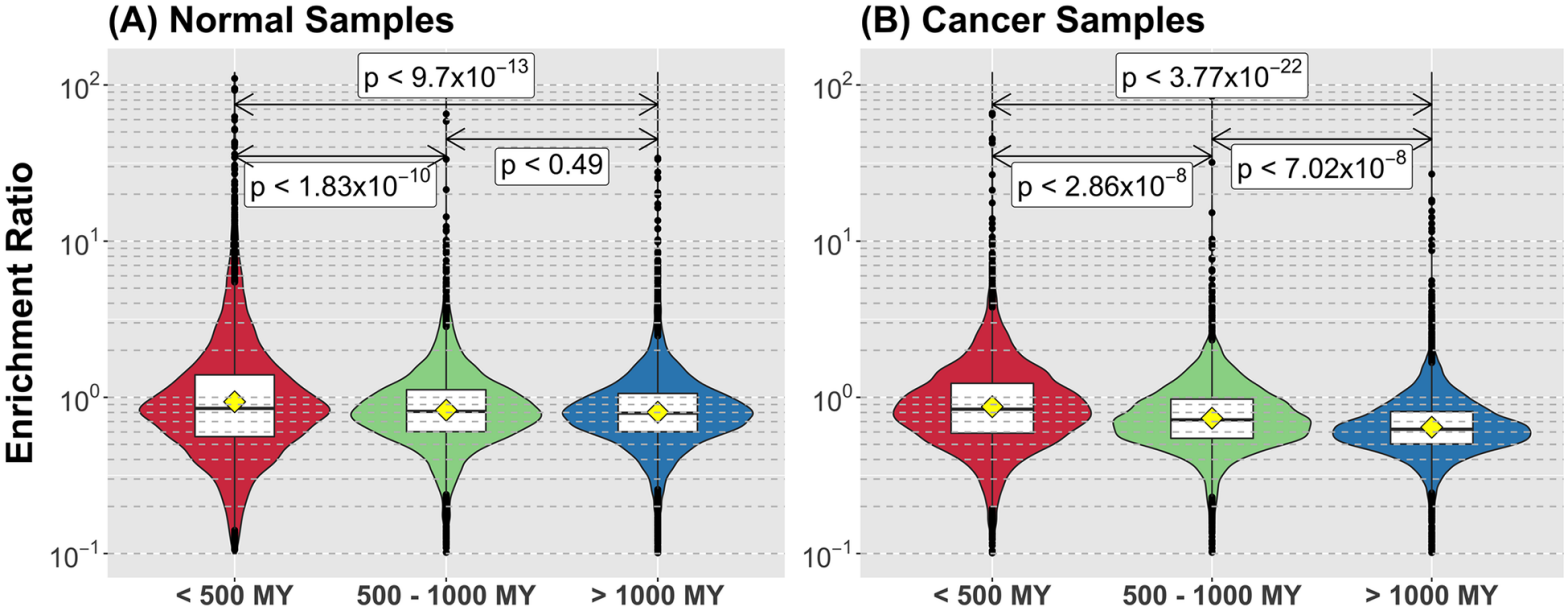
Younger genes are mutated more frequently in both normal and cancer. The Enrichment Ratio is the observed rate of mutation of a gene (in mutations per base-pair) over the expected value according to the null hypothesis of uniform random mutations. We categorized genes in three main age groups, corresponding to post-metazoan (less than 500 MY), metazoan (between 500 and 1000 MY) and pre-metazoan (more than 1000 MY) ages and produced the distribution of Enrichment Ratio for each group. Genes younger than 500 MY old are mutated significantly more frequently in both normal (A) and cancer (B). Also, the frequency of mutation declines as the age of the gene increases. P-values in each case are taken as the maximum between the p-value given by a Tukey's range test between the three groups and a pair-wise t-test comparison.

We tested the first hypothesis by establishing the evolutionary ages of 19,756 human genes by assigning them to gene families according to the Ensembl Compara homology database. We then defined the age of the human member of the gene family as the maximum phylogenetic divergence time between humans and the species represented in the corresponding gene family. Next we examined mutational frequencies as a function of the evolutionary age of each gene in both normal tissue (“normal”) and cancer. In normal tissue, we analyzed the private SNVs from 129 individuals derived from 1000 Genomes Project whole genome sequencing trio data [33]. Under the null model of uniform, random mutation, the frequency of mutation for a given gene is dependent on the length of the gene such that we expect longer genes to accumulate more mutations. When we look strictly at mutational frequency relative to evolutionary age, genes less than 500 MY old were mutated less frequently compared to other age groups (Table A in S1 Text, S2A Fig). Interestingly, when we considered the length of genes relative to their evolutionary ages, genes younger than 500 MY were shorter on average compared to all other age groups (S2C Fig). We controlled for this observation by determining the expected number of mutations per base-pair and then calculated the ratio of observed to expected number of mutations in each gene, generating a fold-change enrichment score. Fig 1A and S2D Fig demonstrate that for their size, genes younger than 500 MY were more likely to be mutated. In addition mutations in genes were 10% less frequent than mutations outside of genes.

We then addressed whether a similar pattern exists in cancer. We looked at 764 samples from of the ICGC (release 19) that had whole genome sequencing with calls for both simple somatic mutations and structural mutations. Under the same null model assumption, genes in cancer cells had 15% less mutation compared to non-gene regions of the genome. Thus cancer recapitulates the pattern seen in normal tissue: mutation occurs predominantly outside of genes, and mutation that occurs within genes is more frequent in genes younger than 500 MY (Table A in S1 Text, S2E Fig).

**Fig 2:**
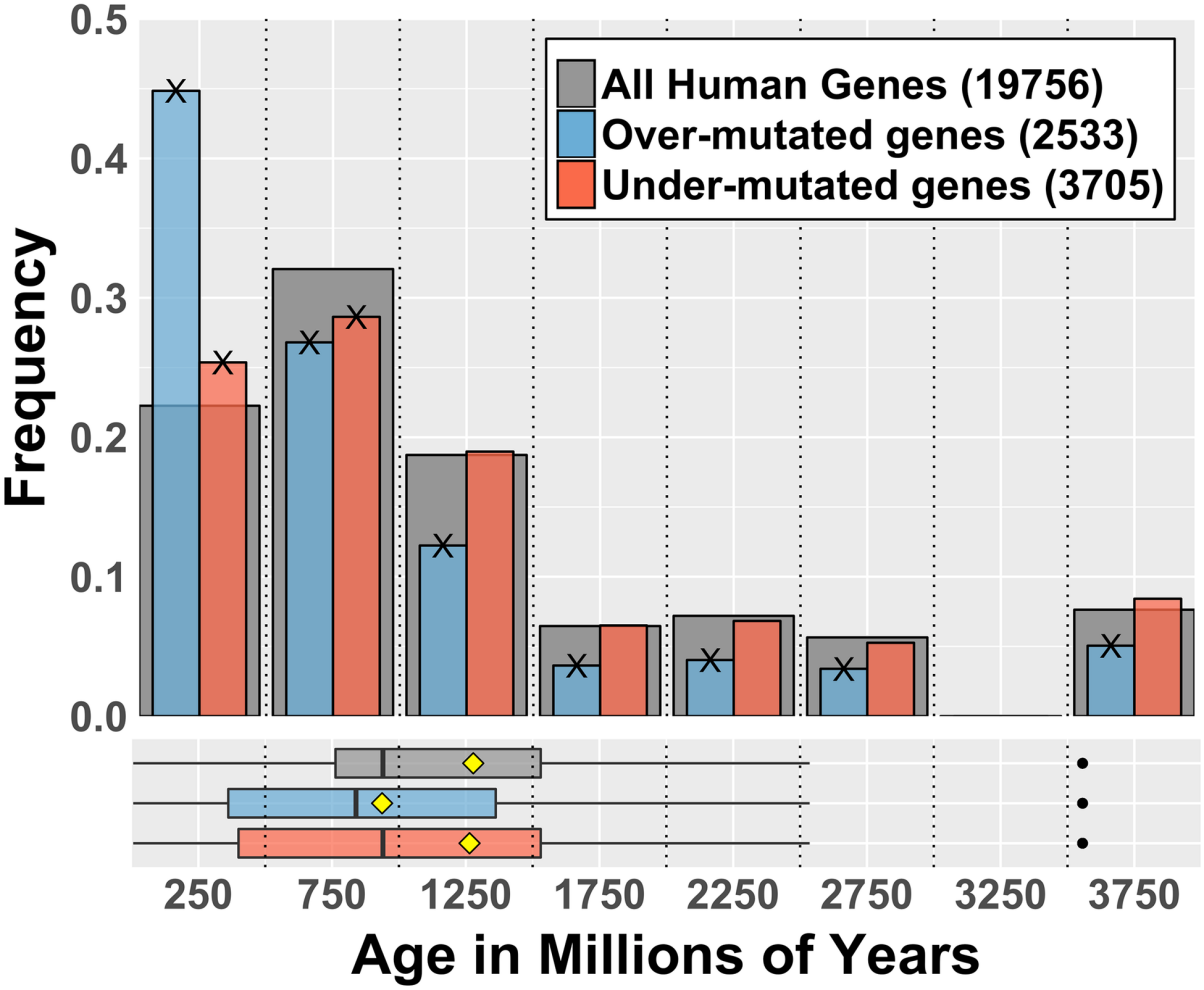
Cancer displays a distinct mutational pattern relative to normal based on the evolutionary age of genes. For each human gene, the expected number of mutations is obtained based on the normal mutation pattern: frequency of normal mutations times the total number of cancer mutations recorded in the data set. According to this, the Enrichment Ratio (ER) is calculated as the ratio of observed cancer mutations and the number of expected mutations in the gene. Over-mutated genes have ER > 1.5; under mutated genes have ER < -1.5. Numbers in legend indicate the size of each gene set. Cross marks (X) on bars tips indicate the enrichment in that category is statistically significant at p < 0.01 according to a bootstrap test taking random samples from the set of all human genes (BSQ < 1%, see Table 1). Boxplots in lower panel show distribution quartiles; black vertical lines are medians, yellow diamonds are means and black dots are outliers.

We also examined the patterns of mutation in cancer relative to what was observed in the normal tissue, which is equivalent to a non-uniform but random distribution in the genome, as shown in Fig 2. We observed that relative to normal, the overabundance of mutation in genes <500 MY becomes even more prominent, while genes older than 1000 MY are typically not over-mutated. The mean age of over-mutated genes is significantly lower than the mean age of all the genes, hence confirming the first hypothesis. Looking in greater detail at the <500 MY age group, we found that cancer indeed appears to profoundly dysregulate mutational processes in genes younger than 500 MY. A greater number of genes in this age group have either more or less than the expected number of mutations (S3 Fig). These results suggest somatic mutation in cancer is preferentially occurring in evolutionarily young genes. But does this mean that genes that drive cancer are evolutionarily young?

**Fig 3:**
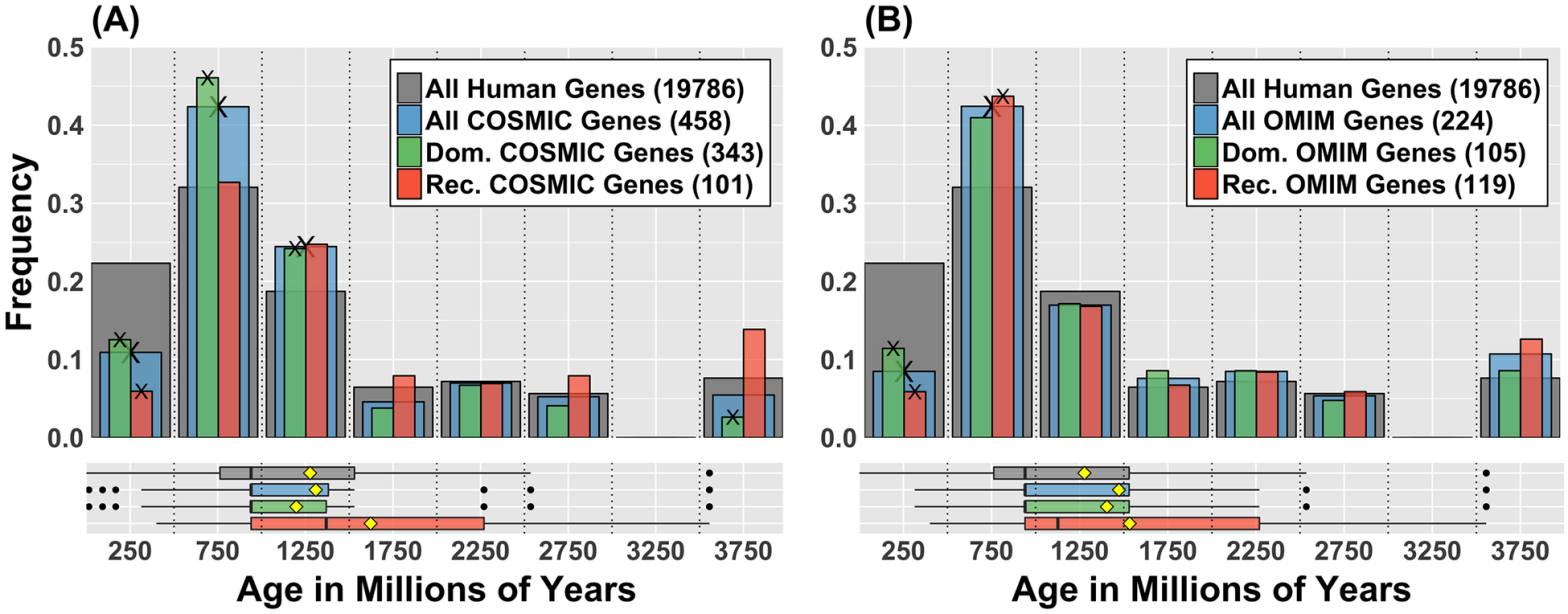
Genes causally implicated in cancer are under-represented among young (<500 MY) genes. (A) Age distribution of dominant (green) and recessive (orange) genes from COSMIC Cancer Gene Census. Grey bars represent the age distribution of all human genes in ENSEMBL, and blue the age distribution of all COSMIC genes. Numbers in legend are the sizes of each gene set. Cross marks (X) on bars tips indicate the enrichment in that category is statistically significant according to Gene Enrichment Score method and a bootstrap test (BSQ < 1%, see Table 1). Accordingly, the second hypothesis predicts that the blue bars should skew to the left with enrichment in both <500 MY and 500–1000 MY. This is not observed. The under-representation of very young genes (less than 500 MY) and the over representation of dominant genes between 500 and 1500 MY are statistically significant. The distinct right skew of the recessive set is also statistically significant (t-test for the difference of the mean with all other sets has p<0.01). This implies that recessive genes are older than expected from random sampling. (B) Similar age distributions for single-gene Mendelian disorders, from the Online Mendelian Inheritance in Man database (OMIM). The general pattern in gene age distributions between dominant and recessive phenotypes observed in cancer, particularly the recessive gene skewness towards old ages and the under-representation of very young genes, is replicated in these gene sets. No notable overrepresentation of dominant genes at moderate ages is detected in this case. The enrichment of cancer genes in such age range is likely associated to breakdown of regulation functions that evolved during the emergence of multicellularity.

To test the second hypothesis that genes that are both frequently and causally mutated in cancer are evolutionarily younger than the emergence of multicellularity as a whole (<1000 MY), we evaluated the evolutionary ages of genes demonstrated to be causally mutated in cancer as compiled by COSMIC in the Cancer Gene Census [31]. In contrast to the model prediction, the general properties of the age distribution of the COSMIC genes did not differ significantly from those of all other human genes (Fig 3A), e.g. as a sample it is representative of the age distribution of all human genes. However, it is clear that there are sub-grouping differences between the two distributions (Table 1). The COSMIC list is enriched with genes having ages that correspond to the development of multicellularity (500–1000 MY), as has been reported previously [20,21], and supports the idea that cancer is at least partially driven by disruption of functions that evolved to achieve multicellular organization. It must be mentioned that this result is also true for the list of genes implicated in single-gene Mendelian disorders (drawn from the Online Mendelian Inheritance in Man OMIM, Fig 3B), suggesting that this is not a feature peculiar to cancer, except perhaps the overrepresentation of genes that evolved with early multicellularity (1000–1500 MY). Interestingly, even though young genes are more likely to be mutated in cancer and normal tissues, both the COSMIC and the OMIM lists are depleted in genes with ages younger than 500 million years (Fig 3).

**Table 1:** Enrichment score for gene age bins of 500 million years for both COSMIC and OMIM genes.

**Age Factor (MY)**	**COSMIC**			**COSMIC Dominant**			**COSMIC Recessive**		
	**Score**^a^	**p-value**^a^	**BSQ**^b^	**Score**	**p-value**	**BSQ**	**Score**	**p-value**	**BSQ**
**< 500**	**0.49**	9.26x10^-5^	**0.02%**	**0.56**	0.0126	**0.02%**	**0.27**	0.00196	**0.02%**
**500–1000**	**1.32**	2.49x10^-16^	**0.02%**	**1.44**	4.38x10^-17^	**0.02%**	1.02	0.118	81.32%
**1000–1500**	**1.31**	1.05x10^-5^	**0.14%**	**1.29**	2.17x10^-4^	**0.88%**	1.32	0.0625	9.96%
**1500–2000**	0.71	0.298	6.94%	0.59	0.15	2.08%	1.23	0.495	39.48%
**2000–2500**	0.97	0.907	81.60%	0.93	1	67.42%	0.96	0.931	81.44%
**2500–3000**	0.93	1	64.70%	0.72	0.438	15.66%	1.4	0.365	23.02%
**3000–3500**	NA	NA	NA	NA	NA	NA	NA	NA	NA
**> 3500**	0.72	0.298	5.40%	**0.34**	0.00145	**0.02%**	**1.82**	0.0395	2.14%
**Age Factor (MY)**	**OMIM**			**OMIM Dominant**			**OMIM Recessive**		
	**Score**	**p-value**	**BSQ**	**Score**	**p-value**	**BSQ**	**Score**	**p-value**	**BSQ**
**< 500**	**0.38**	2.90x10^-4^	**0.02%**	**0.51**	0.177	**0.30%**	**0.26**	7.51x10^-4^	**0.02%**
**500–1000**	**1.32**	5.46x10^-8^	**0.12%**	**1.28**	7.54x10^-4^	4.34%	**1.36**	4.72x10^-5^	**0.54%**
**1000–1500**	0.91	0.781	44.86%	0.92	0.781	60.84%	0.9	0.781	52.28%
**1500–2000**	1.18	0.566	39.40%	1.33	0.566	27.66%	1.04	0.781	71.66%
**2000–2500**	1.18	0.566	37.76%	1.19	0.689	44.62%	1.17	0.689	46.78%
**2500–3000**	0.95	1	77.96%	0.84	1	57.98%	1.04	0.781	71.64%
**3000–3500**	NA	NA	NA	NA	NA	NA	NA	NA	NA
**> 3500**	1.4	0.126	6.40%	1.12	0.781	56.66%	1.65	0.0873	3.86%

Score indicates the enrichment (> 1) or depletion (< 1) of genes in the age category. Scores that are statistically different from 1 as determined by either the p-value or BSQ are bolded.

^a^ Enrichment and p-values were computed as indicated in Zeeberg, et al. [41] where the enrichment score is the proportion of genes within the list from the category divided by the proportion of all human genes that fall into the category. P-values were computer by Fisher’s exact test and then adjusted for multiple comparisons using the method of Benjamini-Hochenberg as implemented in the *p*.*adjust* function of the stats package in R. Scores in bold are have p-values less than 0.05.

^b^ Bootstrap quantile (BSQ) score: for a given gene set, 10,000 random samples of the same size are taken without replacement from the parental list of 19,756 aged human genes and distribution of ages is calculated for each one. The BSQ score for an age group is the percentile quantile in which the actual observed frequency value falls for the corresponding age group in the sampling ensemble. Hence, any BSQ value of less than 1% indicates that the observation is highly unlikely by random sampling.

COSMIC contains 458 genes with different mutational modes of action: those that yield dominant phenotypes and therefore require a single mutant allele (343), and those that give recessive phenotypes (101), requiring that all alleles within the cell be altered. Twelve genes in this list have no clearly defined molecular genetics. It should be noted that the set of dominant genes overlap to a large degree with oncogenes (260 out of 264 genes considered oncogenes [30,40] are dominant), while tumor suppressor genes overlap with recessive genes (70 out of 72 tumor suppressor genes are recessive). However, more than a quarter (122) of the genes in the Cancer Gene Census cannot be classified as either oncogenes or tumor suppressors, although they can still be classified according to their phenotypic expression (dominant or recessive). We observed that the genes with recessive mutations were significantly older relative to all human genes (Fig 3A, Table 1), while genes with dominant mutations were overrepresented at ages that correspond with the emergence of multicellularity. In this respect, cancer resembles single-gene Mendelian disorders (Fig 3B), which also display a difference in the ages of genes with dominant or recessive phenotypes.

### Ancient recessive genes are enriched for DNA repair and cell cycle control

**Fig 4:**
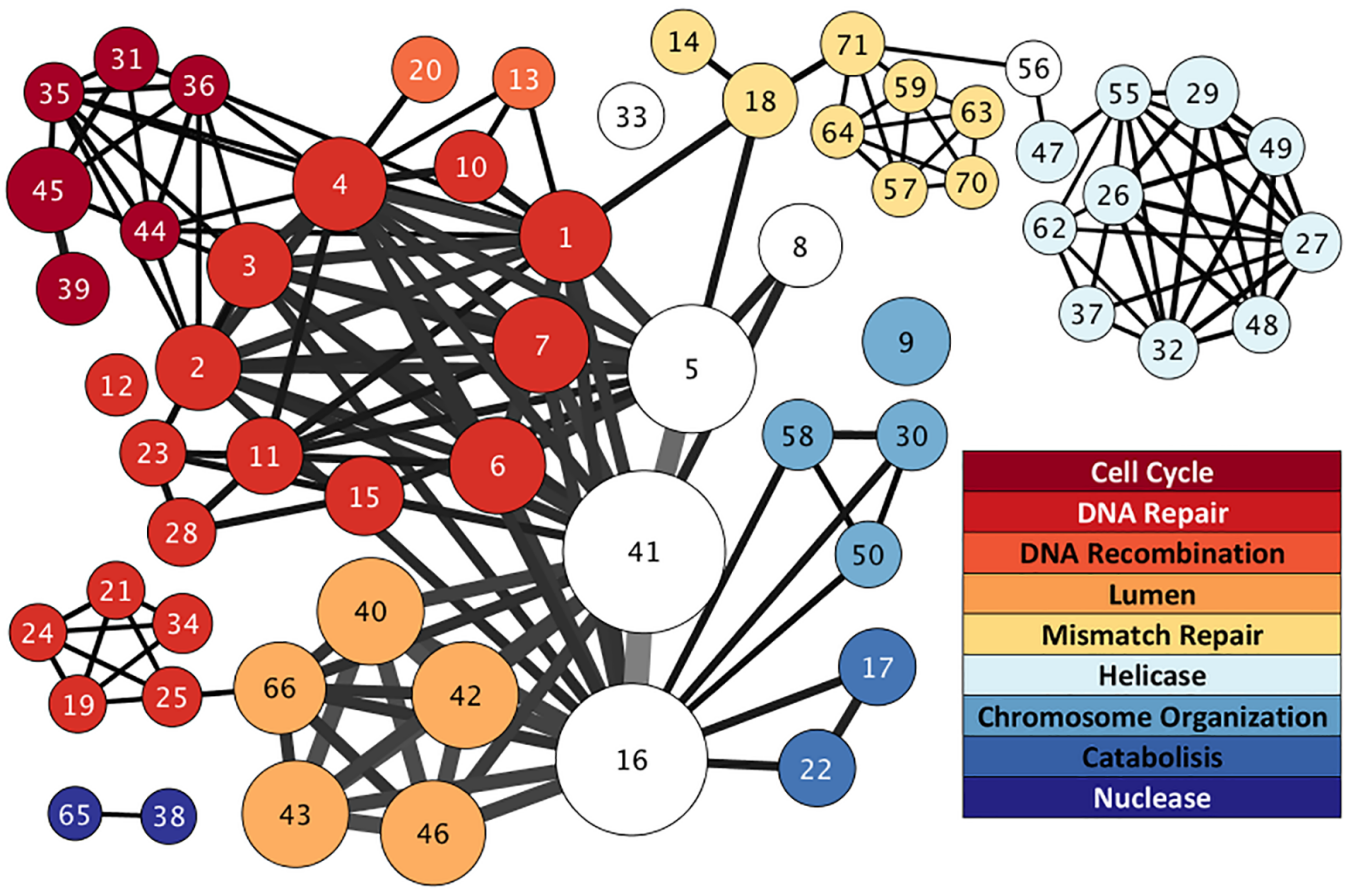
Functional enrichment network of recessive COSMIC cancer genes highlights DNA repair and cell cycle control. Each node in this network represents a group of functionally related genes as returned in DAVID (gene ontology, orthology, functional annotations, etc.). The size of the node represents the number of genes in it. Links between nodes represent gene overlaps between groups, with the width representing the number of genes. Node colors indicate the general functional categories defined in the legend revealing an additional layer of clustering of gene groups. The number in the node indicates the group label as given in S2 Table. Further details of these enrichments for each node are elaborated in S2 Table. For convenience, only nodes with p < 0.002 and FDR < 0.05 are plotted.

The paradox of cancer-causing genes being under-represented in the age bin with the highest frequency of mutation suggests there may be an underlying mechanism that explains the shift in mutational frequency revealed by determining the functions of the dominant versus recessive genes. Functional annotation and enrichment analysis of COSMIC genes using DAVID [36,37] with all human genes as the background list revealed that COSMIC genes with dominant mutations were enriched for transcription factors and transcriptional regulation, immune system development, receptor tyrosine kinases and signal transduction, “stem”-ness and morphogenesis. COSMIC genes with recessive phenotypes were enriched for functions related to DNA repair and cell cycle control (Fig 4); genes with ages older than 950 million years drove such enrichment. The result is so striking that it persists irrespective of the background list used for comparison (recessive cancer genes, all cancer genes, or all human genes). The genes with recessive phenotypes involved in DNA repair focused particularly on double-strand break (DSB) repair and nucleotide excision repair mechanisms. Looking at the evolutionary history of the genes involved in these processes we noted that the non-mutated genes in the same DNA repair pathways, such as REV1, REV3L, POLK, POLH, POLI, POLD1, DMC1, and POLDIP2, are orthologous to genes in bacteria that underlie the adaptive mutation response to stress [7,10,42,43] (see S5 Table for full list of human orthologs). The initiation of the SOS response following the sensing of a double strand break leads to an increase in the rate of both single base-pair mutations and gene amplification events near the DSB as the bacteria switch from high-fidelity replication and repair to error-prone repair. This made us wonder if a similar mechanism was implicated in the patterns of mutations observed cancer.

### Cancers exhibit a molecular signature of stress-induced mutagenesis

In bacteria, the process of adaptive mutation results in a molecular fingerprint in the form of a cluster of SNVs around each DSB[44]. If cancer involves an analogous process, might we find a similar signature? SNV clusters have indeed been reported in cancer[35], but reports thus far fail to consider evolutionary history or genomic distribution relative to a model of uniform, random mutation. This is important because the presence or absence of clusters may be constrained by the interplay between genomic evolution and selection at both organismal and cellular levels.

To address these shortcomings, we used whole genome sequencing data (ICGC release 19) that showed evidence for DSBs. We then evaluated whether or not SNVs clustered in each sample. Out of 764 tumor samples from seven different sites (pancreas, prostate, bone, ovary, skin, blood, and brain), 668 (87.4%) had evidence of SNV clustering. These clusters do not necessarily represent kataegis, defined as 6 or more mutations with inter-mutational distance of 1kb or less [45], but our definition of clustering would catch kataegis events. To evaluate whether the clustering is a peculiarity of somatic mutation in cancer or represents a fundamental process underlying mutation generally, we performed the same analysis on the normal data from 1000 Genomes as a control. Surprisingly, all of the normal samples also had evidence of clustering of SNVs.

**Fig 5:**
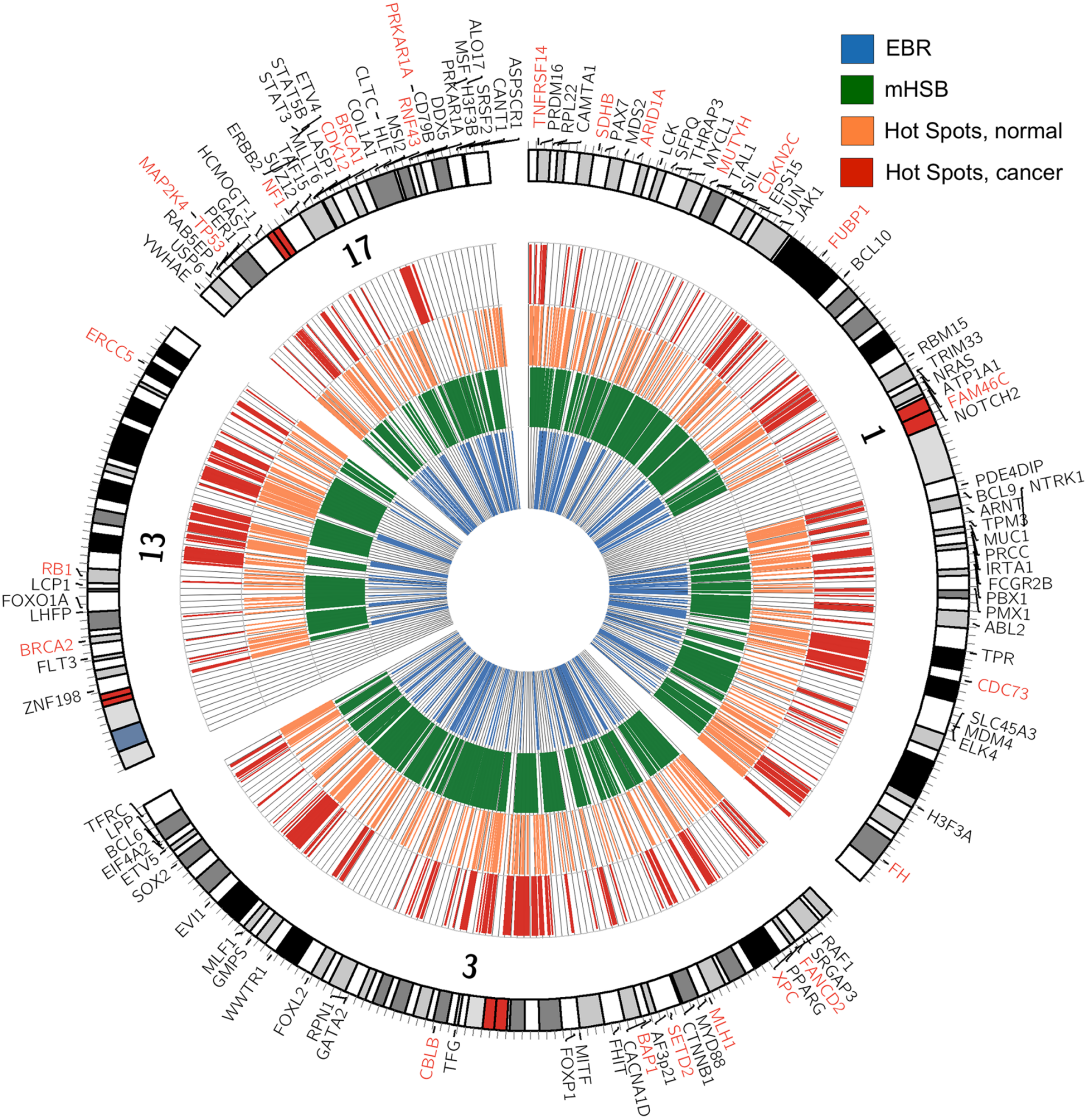
The genomic distribution of SNV clustering differs between normal and cancer. Circos plot showing distribution of SNV clustering for chromosomes 1, 3, 13 and 17. Tracks from inside out are: blue, evolutionarily re-used breakpoint regions (EBR); green, amniote homologous synteny regions (mHSB); orange, hot spots of CM clusters in normal; and red, hot spots of CM clusters in cancer. Outside text track are symbols for COSMIC genes in their corresponding genomic locations. Dominant genes are in black fonts and recessive genes are in red font.

We observed a distinctive difference in the non-random spatial distribution of clusters across the genome in both normal and cancer (Fig 5, S4–S6 Figs), suggestive of “hotspotting” of clusters when considered across samples, e.g. regions of the genome where clusters are more likely to occur (see Methods). We identified regions of cluster hotspotting across samples and examined the evolutionary history of those regions looking at both the ages of the genes in the regions as well as whether or not the regions overlapped evolutionary re-used breakpoint regions (EBR) or amniote homologous synteny blocks (HSB) [46]. EBRs are regions of the genome that have been repeatedly subject to structural rearrangement during amniote evolution. In contrast, HSBs are regions that exhibit not only significant sequence identity, but gene order, across species, and thus represent regions of conserved sequence that have moved as an intact block through genomic evolution. Based on this, we would predict that genes in EBRs would be younger than those in HSBs. Previous work demonstrated that SNPs are more prevalent in EBRs than in HSBs [46]. Therefore, we might expect that cluster hotspots would co-localize with EBRs, be less likely in HSBs, and to be enriched in COSMIC genes.

If human genes are classified as either “metazoan” (less than 1000 MY old) or “pre-metazoan” (older than 1000 MY) we found that the set of pre-metazoan genes overlapped with HSBs and were excluded from EBRs as it might be expected (Table 2). Metazoan genes had the opposite pattern, being excluded from HSBs and enriched in EBRs. Genes on the COSMIC list were preferentially located in HSBs and excluded from EBRs.

**Table 2:** Association of gene age and COSMIC gene status with evolutionarily important regions for genome rearrangement.

Gene set	Overlap with	Odds Ratio	95% Confidence Interval	p-value
**Metazoan genes (< 1000 MY)**	**HSB**	0.6886	0.6503–0.7290	<2.2x10^-16^
	**EBR**	1.096	1.024–1.1.174	8.342 x 10^−3^
**Pre-metazoan genes (> 1000 MY)**	**HSB**	1.452	1.372–1.538	<2.2x10^-16^
	**EBR**	0.9124	0.8521–0.9769	8.342 x 10^−3^
**COSMIC genes**	**HSB**	1.7240	1.409–2.118	3.859 x 10^−8^
	**EBR**	0.7689	0.5965–0.9814	0.03454

Odds Ratios of >1 indicate enrichment, while odds ratios <1 indicated depletion. HSB, homologous synteny region; EBR, evolutionarily re-used breakpoint region.

When considering mutations in normal samples, clustering hotspots co-localized with EBRs and were excluded from HSBs, independently of whether the analysis included all SNV clusters or only those that overlapped genes (Table 3). It is very interesting that COSMIC genes were excluded from hotspot regions in normal samples (Table 4), although genes in that list were slightly more likely to have clusters compared to other genes (odds ratio OR = 1.389, 95% confidence interval CI = 1.124–1.724, p-value = 0.00192). This suggests that cancer is driven by perturbations in parts of the genome that are only slightly more prone to mutation under normal circumstances. Additionally, cluster hotspots in normal samples overlapped younger genes (1157 MY in hotspots versus 1380 MY outside of hotspots, t-test = -9.8927, df = 2825.4, p-value < 2.2x10^-16^). The genes showing evidence of SNV cluster hotspotting were enriched in olfactory receptors, glycoproteins, C-type lectins, oxioreductase functions, steroid metabolism/cytochrome P450 function, serine proteases, oxygen binding, and chromoproteins.

**Table 3:** Co-localization of cluster hotspots with evolutionarily important regions for genome rearrangement in normal peripheral blood.

Normal	Hotspots in	Odds Ratio	95% Confidence Interval	p-value
**Whole Genome:**	**HSB**	0.3053	0.3012–0.3093	<2.2x10^-16^
	**EBR**	1.135	1.119–1.152	<2.2x10^-16^
**Overlapping Genes:**	**HSB**	0.3256	0.3183–0.3331	<2.2x10^-16^
	**EBR**	1.757	1.715–1.800	<2.2x10^-16^

The comparison was run looking at private SNVs (determined from trio comparison) clustering across the entire genome as well as clustering that only overlapped genes. HSB, homologous synteny region; EBR, evolutionarily re-used breakpoint region.

**Table 4:** Overlap of COSMIC genes with cluster hotspots (i.e. clustering of clusters) in both normal peripheral blood and tumors based on clusters that overlap genes.

Category	Odds Ratio	95% Confidence Interval	p-value
**Normal**	0.7666	0.7189–0.8172	<2.2x10^-16^
**Cancer**	0.3246	0.2897–0.4034	<2.2x10^-16^

For mutations observed in cancer samples, clustering hotspots typically overlapped with younger genes (mean gene age in hotspots was 1035 MY old versus 1360 MY old for genes outside of hotspots, t = -10.412, df = 1007.8, p<2.2.x10^-16^). Unlike the normal data, the overlap of hotspots with either HSBs or EBRs depended on the clusters included in the analysis. Genome-wide, hotspots were excluded from HSBs, but among clusters that overlapped genes, there was no exclusion or enrichment (Table 5). When we considered only clusters that overlap genes, the hotspots were more prevalent in EBRs. However, hotspots were preferentially excluded from EBRs when we analyzed all clusters across the genome. As with normal data, COSMIC genes were excluded from cluster hotspots in cancer (Table 4). However, COSMIC genes were more likely to contain clusters by almost 2-fold (OR = 1.873, 95% CI: 1.546–2.272, p-value = 4.386x10^-11^) compared to other genes. This suggests that cluster hotspots are driven by constraints placed on the genome by evolution, and the mechanism of clustering allows mutation to occur in genomic space that is usually off-limits evolutionarily. The functional enrichment of genes that overlap clusters in hotspots was also different in cancer. Hotspots in cancer co-localized with genes that are enriched for extracellular glycoproteins, G-protein coupled receptors especially olfactory receptors, cell-cell adhesion, molecular species intrinsic to the plasma membrane, Ig- and EGF-like domains, ligand gated ion channels, membrane attack complex component/perforin, complement 9, cadherin, Sushi domains, potassium channels, Kazal proteinase inhibitors, fibronectin III, glycosylation, glycoproteins, the machinery to hydrolize and excrete proteins, and finally MHCI and MHCII.

**Table 5:** Co-localization of cluster hotspots with evolutionarily important regions for genome rearrangement in cancer genomes.

Cancer	Hotspots in	Odds Ratio	95% Confidence Interval	p-value
**Whole Genome:**	**HSB**	0.5443	0.532–0.5569	<2.2x10^-16^
	**EBR**	0.8291	0.8078–0.851	<2.2x10^-16^
**Overlapping Genes:**	**HSB**	0.9598	0.9164–1.005	0.08166
	**EBR**	1.4354	1.370–1.504	<2.2x10^-16^

The comparison was run looking at all clusters as well as only those clusters that overlap genes. HSB, homologous synteny region; EBR, evolutionarily re-used breakpoint region.

**Fig 6:**
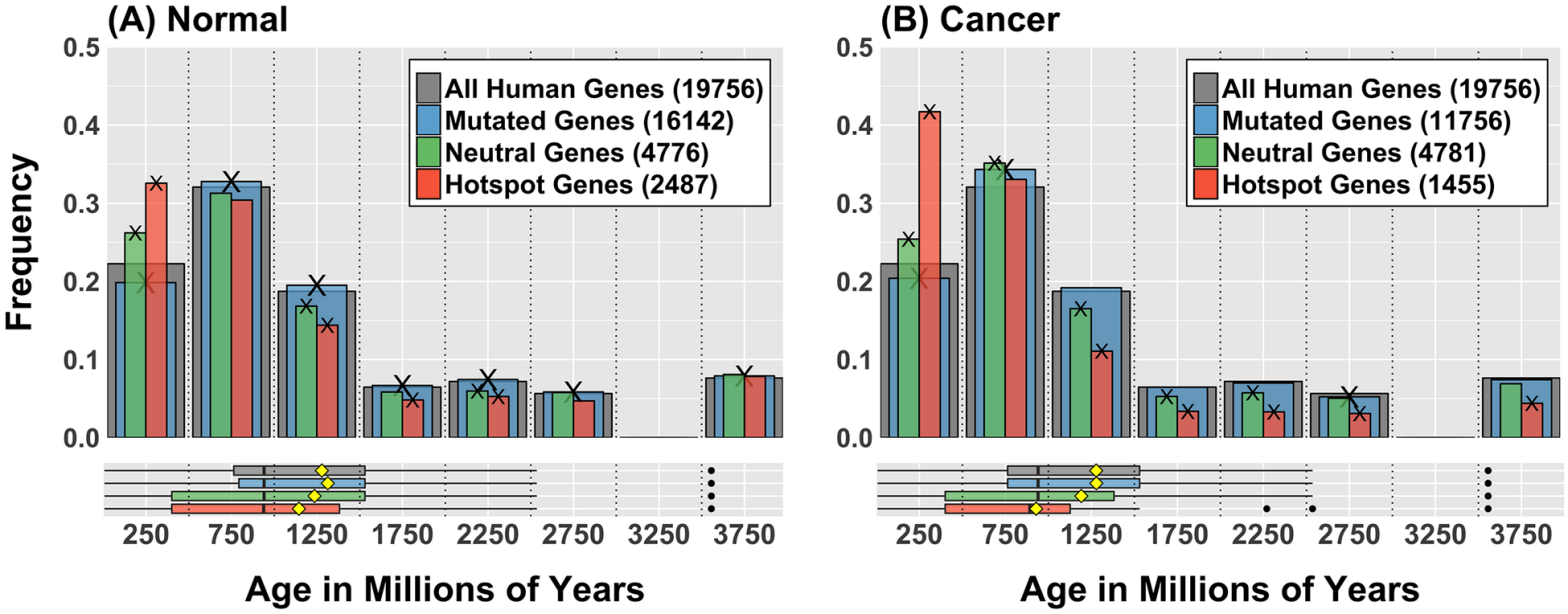
Mutational pattern in young genes is characterized by hot-spotting. (A) Age distribution of all genes mutated in normal samples data (blue), genes that have neutral level of mutation, as expected from a uniform random distribution (green) and genes in hotspots (orange). Grey bars represent the age distribution of all human genes. Numbers in legend are the sizes of each gene set. Cross marks (X) indicate the enrichment in that category is statistically significant according to a bootstrap test (BSQ < 1%, see Table 1). Boxplots in lower panel show distribution quartiles; black vertical lines are medians, yellow diamonds are means and black dots are outliers. (B) Equivalent plots for cancer data (ICGC release 19). In both plots when we observe the age distribution of genes involved in hotspots (orange), a very large proportion of them are very young (less than 500 MY). This suggests that the mutational activity that produces hot-spotting in the genome is preferentially hitting younger genes in spite of the fact that they are generally under-represented in the sets of all observed mutations.

Interestingly, the hotspot enrichment of young genes was even more evident when we compared the age distribution of mutated genes for both normal and cancer data (Fig 6). In this case we observed that younger genes are profoundly over-represented in the set of genes that overlap hotspots despite the fact that that same group of genes is significantly under-represented for general mutations. In combination with the fact that young genes are markedly shorter, these observations suggest that the pattern of mutations in young genes is targeted, possibly under some form of control, and strongly subjected to whatever mechanism generates cluster hotspots in the genome.

## Discussion

Our work highlights the deep evolutionary roots of cancer and the importance of the evolutionary history of the genome in mutational processes driving oncogenesis. Previous studies of cancer gene ages rely on sparse phylogenetic trees [20,21], and therefore lack the power to resolve older genetic history. Our investigation is able to probe earlier epochs. Our study confirmed the earlier observation of an abundance of genes considered causal in cancer at ages that span the evolution of multicellularity, but it also revealed that many cancer-causing genes are much older than previously appreciated. Our analysis shows there is a complex interplay between the evolutionary history of the genome and the somatic processes shaping the mutational landscape of cancer. We demonstrated that mutational processes in both normal and cancer cells are more common in evolutionarily young genes and regions of the genome repeatedly used for structural rearrangement. Mutation was generally excluded from regions of the genome that have been conserved both in sequence and linear order over large stretches of DNA through evolutionary time, where genes considered casual in cancer are likely to be located. Thus, our data demonstrate there are regions of the genome that appear to be hotspots for mutation and other regions that seem to be protected, probably through a combination of differential repair mechanisms and protection against mutations. Hotspot regions are more likely to overlap genes that are evolutionarily young. The genomic instability seen in cancer has to operate against this pre-existing background, i.e. it is constrained by the evolutionary history of the genome. On the face of it, we might therefore expect mutational hotspots in cancer to a) affect genes known to be frequently and causally mutated in cancer and b) for causally mutated genes to be evolutionarily young. Intriguingly, our analysis refuted both these predictions: genes that are frequently mutated and causal in cancer are both older and excluded from these hotspots, although they show a 2-fold enrichment in mutational clustering compared to other genes. Why?

The answer would seem to lie in the inherent conflict between different levels of selection that operate in a multicellular organism, where, particularly during development, there is selection both at the cellular level and at the organismal level. Many of the genes that are causal in cancer have significant roles in development [47–51]. Selection at the organism level will remove mutations that might be tolerated at the cell level but cannot be tolerated by the organism as a whole, effectively protecting the affected genes from hotspotting over time. But since cells are more likely to survive if mutations happen in a coordinated mutational burst [52], these genes are not necessarily protected from the mechanisms of cluster formation, and the resulting mutational clusters can be recovered if the selection pressure moves from both organismal and cellular levels to only the cellular level. If cancer is a reversion to single-cell behavior, then the selective pressure on cancer cells move closer to a state dominated by cellular level selection. It is no surprise then, if in response to an insult or stress, a cancer cell adopts a survival strategy that might ultimately prove detrimental to the organism. One such strategy is to reactivate the ancient prokaryotic process of stress-induced mutagenesis, which relies on low-fidelity breakage-induced replication to generate coordinated clusters of mutational events that effectively increase the chances of adapting to the stressful environment through evolution. Activating such a mechanism might also increase the chances of succesful mutation in genomic regions where the probability of mutation is low because of the evolutionary constraints of the genome. These are the regions where many genes important in oncogenesis reside.

The functional annotation of old recessive cancer genes led to the hypothesis that stress-induced mutation plays a role in genomic instability in cancer and the mutational clusters seen in cancer represent the molecular signature of a conserved stress-induced mutagenesis response. The genes in humans that are orthologous to the error-prone polymerases that mechanistically drive the stress-induced mutation response in bacteria have become specialized DNA polymerases for translesion synthesis (TLS) employed during replication by-pass of DNA damage [42]. These polymerases recognize specific types of DNA damage and faithfully replicate the damaged DNA (for example incorporating a C when encountering O-methyl-guanine) but exhibit orders of magnitude less fidelity against undamaged DNA [42]. Thus, for DNA damage incurred or persisting into S-phase, these so-called error-prone DNA polymerases will be employed during replication leading to an overall up-tick in single base mutations. In normal cells, the tightly controlled regulation of DNA repair with cell cycle prevents the propagation of the vast majority of these TLS mutations into the next cell division by halting the cell cycle and allowing time for mismatch recognition and repair, or in extreme cases, generating an apoptotic response, at least in somatic cell lineages.

In bacteria the stress-induced mutation response leaves behind a molecular signature that can be detected in the form of SNV clusters around DSBs [44]. We observed analogous clusters in the whole genome sequences of human tumors as well as in normal peripheral blood, the latter reflecting the *de novo* mutations arising during meiosis or early embryonic development. These clusters are not randomly distributed across the genome, with a distribution that differs according to whether they come from normal or cancer samples, further supporting that idea that cancer is mutating a different subset of genomic space compared to normal tissue. The data from normal samples imply that there may be a developmental regulation of mutational bursts outside of the well-recognized somatic hyper-mutation processes in the immune system. This is supported by recent work on the rate and timing of mutations in the germline [53]. Additionally, recent work by Francioli et al suggests that there is a role of TLS in the generation of *de novo* mutations in the germline [54]. In their study, they observed that clusters of mutations were enriched in C > G transversions but not in the sequence contexts recognized by APOBEC relative to non-clustered mutations. They postulate that they are the result of error-prone TLS [54].

In cancer, the role of TLS in the generation of genomic instability has been recognized but attributed to oncogene-induced replication-stress, not the induction of a programmed mutational response [55]. The abrogation of the link between DNA damage and cell cycle by eliminating efficient activation of cell cycle checkpoints or altering the function of some but not all DNA repair pathways, leads to persistent DNA damage, TLS employment, and an increase in TLS introduced errors that survive to the next round of replication. Recent work in yeast on mutagenic breakage-induced replication demonstrated both a reliance on TLS as well as a resulting mutation pattern that resembles the phenomenon of kataegis seen in cancer [56,57], suggesting an additional mechanism by which TLS could be involved in the generation of mutational clusters. The decrease in DNA repair capacity of the cancer cell per unit time may also play into the role of APOBEC in generating clusters of mutations in cancer [35,58] through increasing the amount of single-single strand DNA substrates available in the genome. However, only roughly half of the clusters identified by Roberts, et al. had a sequence context suggestive of APOBEC or AID activity [35]. Additionally, TLS is thought to play a role in the C > G transversions in APOBEC driven clusters through by-pass of abasic sites as a result UNG driven repair of the resulting uracil.

Altogether our results on the age of the recessive genes, the homology to the proteins involved in stress-induced mutation in bacteria to non-mutated genes in DNA repair and cell cycle pathways in humans, and the observation of the molecular signature of stress-induced mutation in human tumors are strong evidence for the restoration of a stress-induced mutational response in somatic cells. Our analysis supports the idea that the stress induced mutational program remains functional but has become cell-lineage constrained. Based on our analysis we propose that, in multi-cellular organisms, the restriction of mutational processes that promote evolution in the germline and the immune system was brought about by re-wiring the input for the mutational response to be a developmental signal, rather than a cellular stress signal. This in turn suggests epigenetic control. A variety of conditions, such as chronic inflammation, may lead to microenvironments where the epigenetic regulation that keeps the mutational program under developmental and lineage control are altered, allowing somatic cells inappropriate access to a stress-induced mutational response. Thus, we propose stressed-induced mutation as a hallmark of cancer reflected by genomic instability.

Our results have important implications for the clinical management of cancer. There is already evidence that TLS polymerase expression contributes to both intrinsic and acquired resistance to genotoxic therapies [59–67]. However, the mechanism of stress-induced mutagenesis would predict a role for TLS activity in resistance to a wide range of therapies, including targeted therapies. The current paradigm for understanding therapeutic resistance contends that intracellular heterogeneity leads to multiple, clonal subpopulations with *a priori* different susceptibilities to treatment. Treatment creates a bottleneck resulting in clonal selection. This selection is inferred from the observation that mutational events that have become fixed in the population may dramatically alter their frequency following treatment [68–70]. The surviving clones then re-establish their diversity after the fact because of ongoing instability. Resistance to therapy arises either because it existed *a priori* and survives the clonal sweep or it develops as the population re-diversifies at the cellular level. However, the phenotype of stress-induced mutation would predict that the bottleneck itself is the primary driver of intercellular genomic diversity leading to the acquisition of resistance. Furthermore, both clonal selection and regeneration of intercellular diversity occur simultaneously. If we accept that tumor formation occurs over years in most cases, then the rapid and almost universal acquisition of resistance to inhibitors of the BRAF V600E mutation is suggestive that this is indeed occurring [71]. The rapidity with which resistance to an effective therapy is acquired would likely depend on how robustly a given tumor has activated the stress-induced mutation program. Treatment dynamics are likely to be very important in minimizing the impact of stress-induced mutation on tumor progression, with both the intensity and duration of exposure playing a role. We hypothesize that there is a threshold effect of stress induction below which tumor cell fitness is compromised but elevated mutation is not induced. Thus, lower doses given more frequently may be more effective at controlling cancer progression in the long run. This is supported by a recent study showing that intermittent dosing of patient-derived xenografts of BRAF V600E mutant melanoma results in a failure to reach lethal drug resistance, even when the cumulative dose meets or exceeds that received on a continuous dosing regimen where all tumors acquired lethal drug resistance [72]. Similarly, in newly diagnosed multiple myeloma, patient outcomes were the same or better with lower toxicities on a regimen of Lenalidomide plus low-dose dexamethasone [73]. In preclinical models of breast cancer, adaptive therapeutic treatment, where an initial large dose of paclitaxel is used to drive the tumor growth rate to plateau followed by regular doses that were then adjusted based on the change in tumor size, led to long term stabilization of tumor growth and increased survival [74]. This suggests a fundamental switch in treatment paradigm from maximum tolerable dose to minimum efficacious dose and the use of metronomic or adaptive therapy strategies, and from ‘cure’ to management of cancer as a chronic disease.

In conclusion, our analysis suggests that the observed phenotype of evolvability in cancer is driven by re-activation of an evolutionarily ancient stress-induced mutational response. Understanding the parameters of this response will be key to maximizing the effectiveness of cancer treatment.

## Supporting information

S1 Text Includes additional information about methods, analysis and Tables A, B, and C. (DOCX)

S1 Fig Distributions of ages for Ensembl/Compara, HOGENOM and NCBI HomoloGene homologies. HomoloGene fails to reveal tree nodes corresponding to events of early evolution (older than 1500 MY), in turn giving a relative over-representation of resent events (less than 500 MY). The evolutionary time spanned by HomoloGene is later than the evolution of multicellularity.
(TIFF)

S2 Fig Genes younger than 500 MY are more frequently mutated after controlling for gene length. Frequency of Gene Mutation according to gene age. The distribution of values of mutation frequencies for each age group is estimated and shown as vertical violin and box plots. Horizontal lines are the median; circle is the mean and black dots are distribution outliers in each case. Vertical axis is in log scale. Corresponding plots are shown for both normal (A) and cancer data (B). In both cases it is evident that genes the first age bin (age < 500 MY) are typically mutated less frequently than the rest. (C) Distribution of gene lengths according to age group membership. Young genes are typically shorter than other genes. Frequency of gene mutation normalized by gene length for both normal (D) and cancer data (E) shows that young genes are more likely to be mutated. Groups were compared via ANOVA followed by Tukey’s Post-Hoc test to determining which relationships were driving the partitioning of variation. In normal (D), the <500 MY age bin is more frequently mutated compared to all other age bins (for all pair-wise comparisons, p<2.2x10^-16^). In cancer (E), the <500 MY age bin is more frequently mutated compared to all other age bins (for all pair-wise comparisons, p<2.2x10^-16^). Additionally, the 500–1000 MY age bin was more frequently mutated compared to 1000–1500 MY (p = 10^−7^), 1500–2000 MY (p = 3.2x10^-6^), and 2000–2500 MY (p = 3.6x10^-4^).
(TIF)

S3 Fig Cancer displays a distinct mutational pattern relative to normal based on the evolutionary age of genes. For each human gene, the expected number of mutations is obtained according to the normal mutation pattern: frequency of normal mutations times the total number of cancer mutations. The Enrichment Ratio (ER) is the ratio of observed cancer mutations and the number of expected mutations in the gene. We define six different gene categories according to the level of enrichment and produce age distributions. Unexpected mutated genes are those genes that are never normally mutated but are mutated in cancer; Severely over-mutated genes are those with over 10 times more mutations in cancer than normal (ER>10); Moderately over-mutated genes are mutated 1.5 to 10 times more in cancer than normal (10>ER>1.5); Unaffected genes have more or less the same number of mutations in cancer than normal (1.5>ER>0.67); Moderately under-mutated genes are mutated up to ten times less than normal (0.67>ER>0.1); and Severely under-mutated genes are mutated more over 10 times less than normal, including a few genes that normally mutate but are never found mutated in cancer. Numbers in legend are the sizes of each gene set. Cross marks (X) on bars tips indicate the enrichment in that category is statistically significant according to a bootstrap test.
(TIFF)

S4 Fig Circos plot showing distribution of SNV clustering by chromosome. Chromosomes 1 to 8.
(TIF)

S5 Fig Circos plot showing distribution of SNV clustering by chromosome. Chromosomes 9 to 16.
(TIF)

S6 Fig Circos plot showing distribution of SNV clustering by chromosome. Chromosomes 17 to 22 and X.
(TIF)

S1 Table Evolution of biological functions as determined by gene function enrichment. (XLSX)

S2 Table Details for functional enrichment network of recessive COSMIC cancer genes. The network plot for the enrichment is shown in Fig 4. Each node in the network represents a group of functionally related genes as returned in DAVID (gene ontogeny, orthology, functional annotations, pathways, etc.). An additional level of clustering is represented by node colors defined in this table, revealing general functional associations of gene groups. Enrichment scores for each of these categories are shown.
(XLSX)

S3 Table Human orthologs to *E*. *coli* genes involved in stress-induced mutation. Human orthologs of *E*. *coli* genes identified by Al Mamun, et al. [43].
(XLSX)

S4 Table List of donors used in analysis for both ICGC and 1000 genomes data. (XLSX)
