# Supplementary material for: Ancient genes establish stress-induced mutation as a hallmark of cancer
Source: PLoS One. 2017 Apr 25;12(4):e0176258. doi: 10.1371/journal.pone.0176258 (PMC5404761; doi:10.1371/journal.pone.0176258)
Supplement: S1 Text — (DOCX) [file pone.0176258.s001.docx]

# Supporting Information

## Differences in evolutionary gene ages inferred from three databases of homologous gene families

Given that our gene ageing method is contingent on the definition of homology, more accurate gene family builds will produce better estimates of gene ages. Human gene families in Ensembl Pan-Taxonomic Compara [1] (EC, 19,780 genes), NCBI HomoloGene [2] (HG, 18,304 genes) and HOGENOM [3] (17,086 genes from the nucleotide database) were collected and assigned evolutionary ages according to the TimeTree [4] divergence time of the last common ancestor of each gene family.

Discrepancies in gene ages produced by the different databases are, in large part, due to differences in their approaches to determine gene homologies across species. All methods to identify orthologous genes across multiple species start with a protein sequence similarity search with more or less sophistication. For instance, protein similarity and DNA sequence can be combined together and information about genetic synteny (interspecies gene order and orientation correspondence) could be used. In this way, different similarity cut-offs are typically considered in order to determine the homology between genes of different species. But gene duplications and losses occurring during speciation events can complicate the reconstruction of the gene families. Phylogenetic trees that result from a group of homologous genes may not necessarily agree with the species phylogeny. Thus refined methods use tree-reconciliation techniques intended to harmonize the gene family phylogenetics consistently with species phylogeny.

Both HomoloGene and Ensembl Compara do some form of protein and DNA similarity and synteny-driven sequences matching. On the other hand Ensembl Compara and HOGENOM implement some level of reconciliation with species trees, while HomoloGene uses a crude method that consist of comparing protein sequences of closer species first in order to build family trees. In Ensembl Compara, trees from multiple alignments in protein-coding genes and ncRNA genes are built independently, producing sets of trees further analyzed to extract orthologues and paralogues, and reconciled with species trees, allowing the calling of duplication events and produce the most likely tree from the sets. Also Ensembl Families and Protein Trees undergo a step of stable gene identification mapping that allows for tracking the update of a tree or a family across database releases.

Original identifiers (Entrez Gene for HomoloGene, Ensembl for Compara, and Uniprot for HOGENOME) were mapped with each other according to current identification table (release July 29, 2014).

The data from HOGENOM was discarded because the last available update is from December 2011, making gene identifiers out of date with current builds of HG and EC and thus introducing unavoidable artifacts in downstream analysis. Mapping into current Entrez Gene ID we picked up 18,214 unique gene calls from HG and 17,981 from EC. Of these, 16,852 genes are in the overlap between the two databases. The correspondence of gene age between the databases has a Pearson’s correlation coefficient of 0.54 with a significance p < 10^-5^. Therefore pair comparisons are not likely purely random fluctuations. The distribution of ages per gene by database (S1 Fig) suggests that the lack of correspondence is due to a set of genes described as very old in EC but not in HG, and alternatively, a set of genes deemed very young in HG, but not in EC. The distributions of ages for different databases are statistically independent (general K-S test, D= 0.4897, k_s_ = 44.952092 and p < 10^-7^.) The median of the difference between EC and HG for each gene is 497.65 million years, with an interquartile range of 787.12 million years. There are 3988 genes (23.66%) of the list that have a difference greater than one billion years.

**S1 Fig:** **Distributions of ages for Ensembl/Compara, HOGENOM and NCBI HomoloGene homologies.** HomoloGene seems to fail to reveal tree nodes corresponding to events of early evolution (older than 1.5 billion years), in turn giving a relative over-representation of resent events (less than half a billion years). The evolutionary time spanned by HomoloGene is posterior to the evolution of multicellularity.

When the identity of the genes with extreme differences in age is examined, it is evident that they correspond to human paralogs. A functional annotation of 2079 genes with age differences of more than 500,000 years was done using DAVID [5] (and Ensembl Gene ID), revealing enrichment for zinc finger and KRAB regions. Further exploration of the enrichment clusters showed that the functions enriched are related to mitosis, intracellular membranes, zinc fingers and transcriptional regulation, and detoxification by cytochrome p450 or s-glutathione transferases. All these classes are ancient functions for which there have been multiple duplications and radiation events during evolution and for which there can be high sequence similarity between paralogs as well as between orthologs, making it difficult to establish an accurate phylogenetic tree.

Based on this analysis we considered that Ensembl Pan-Taxonomic Compara was the best option for determining homology, and computed gene ages based on it for the rest of our analysis in this paper.

## Gene Ageing Methods, additional details

The age of a human gene is defined by the divergence time of the last common ancestor of all the species in the human lineage that share gene homologies to such gene. Strictly speaking the age of a common ancestor in an evolution tree is always larger than the divergence time between each species branching out from it, therefore this measure provides an estimate of the minimum expected age of the gene.

We implemented a branch-length method for comparing paralogous human genes. In a gene family tree the gene with the shortest distance to the root, measured in terms of the average rate of substitutions per nucleobase, is the one that is most similar to the ancestral sequence and therefore the oldest gene in the family tree. Similarly, the gene with the largest distance to the root is least similar to the ancestral sequence and thus the newest gene. Assuming an approximately constant rate of evolution across the tree, the ages of intermediate genes are directly proportional to the distance from that gene to the root of the tree. In this way, for the *j*-th gene family, if *L_j_* is the maximum distance from the root to the leafs, *D_i_* is the distance from the root to the *i-th* tree node, and *F_j_* is the gene family age, then the length-estimated age of the gene at node *i* is given by:

A_i_ = $\left( 1-\frac{D_{i}}{L_{j}} \right)*F_{j}$

This length-based method should allow us to deduce age differences between gene paralogs. Unfortunately this approach relies too heavily on the assumption of constant evolution rate and has complications in addressing gene duplication events, gene losses and horizontal gene transfers that complicate its implementation for batch analysis. As such, this method produced differences larger than 150MY in 107 genes and the distributions of age were identical to the original method except in genes of age 300-500MY, in which case some genes became slightly younger, not older. In view of this and the fact that our interest is to investigate the general properties of the evolution of functions in the human genome, we did not use the length-based method in downstream analyses. This method may be in future work, particularly for specific gene families that could be analyzed in a supervised manner and for which the evolution pattern along speciation events is relatively known.

## Gene Age and Mutational Frequency

**Table A:** Distribution of the frequency of mutation for genes as a function of evolutionary age in normal genomes (not corrected for gene length).

| **Age Factor (MY)** | **Minimum** | **25^th^ Percentile** | **Median** | **75^th^ Percentile** | **Maximum** |
| --- | --- | --- | --- | --- | --- |
| **<500** | 0 | 3.80 x10^-7^ | 2.28 x10^-6^ | 8.98 x10^-6^ | 6.63 x10^-4^ |
| **500-1000** | 0 | 2.53 x10^-6^ | 8.48 x10^-6^ | 2.47 x10^-5^ | 1.60 x10^-3^ |
| **1000-1500** | 0 | 3.29 x10^-6^ | 1.02 x10^-5^ | 2.62 x10^-5^ | 9.92 x10^-4^ |
| **1500-2000** | 0 | 3.42 x10^-6^ | 1.02 x10^-5^ | 2.44 x10^-5^ | 5.85 x10^-4^ |
| **2000-2500** | 0 | 3.16 x10^-6^ | 9.87 x10^-6^ | 2.59 x10^-5^ | 2.76 x10^-3^ |
| **2500-3000** | 0 | 2.91 x10^-6^ | 7.97 x10^-6^ | 2.13 x10^-5^ | 7.95 x10^-4^ |
| **>3500** | 0 | 3.42 x10^-6^ | 8.47 x10^-6^ | 2.19 x10^-5^ | 5.97 x10^-4^ |

**S2 Fig:** **Genes younger than 500 MY are more frequently mutated after controlling for gene length.** Frequency of Gene Mutation according to gene age. Seven age groups were defined and the distribution of values of mutation frequencies for each set was estimated and shown as vertical violin and box plots. Horizontal lines are the median; circle is the mean and black dots are distribution outliers in each case. Vertical axis is in log scale. Corresponding plots are shown for both normal (A) and ICGC (release 19) cancer data (B). In both cases it is evident that genes the first age bin (age < 500 MY) are typically mutated less frequently than the rest. (C) Distribution of gene lengths according to age group membership. Young genes are typically shorter than other genes. Frequency of Gene Mutation normalized by gene length for both normal (D) and cancer data (E) shows that young genes are actually more likely to be mutated. Groups were compared via ANOVA followed by Tukey’s Post-Hoc test to determine which relationships were driving the partitioning of variation. In normal (D), the <500 MY age bin is more frequently mutated compared to all other age bins (for all pair-wise comparisons, p=0.0000000). In cancer (E), the <500 MY age bin is more frequently mutated compared to all other age bins (for all pair-wise comparisons, p=0.0000000). Additionally, the 500-1000 MY age bin was more frequently mutated compared to 1000-1500 MY (p=0.0000001), 1500-2000 MY (p=0.0000032), and 2000-2500 MY (p=0.0003561).

**Table B:** Distribution of the frequency of mutation for genes as a function of evolutionary age in cancer genomes (not corrected for gene length).

|  | **Age Factor  (MY)** | **Minimum** | **25th Percentile** | **Median** | **75th Percentile** | **Maximum** |
| --- | --- | --- | --- | --- | --- | --- |
| **ICGC Release 19** | **<500** | 0 | 5.06 x10^-7^ | 2.02 x10^-6^ | 6.75 x10^-6^ | 6.41 x10^-4^ |
|  | **500-1000** | 0 | 2.36 x10^-6^ | 6.91 x10^-6^ | 1.92 x10^-5^ | 1.64 x10^-3^ |
|  | **1000-1500** | 0 | 2.87 x10^-6^ | 7.25 x10^-6^ | 1.80 x10^-5^ | 1.17 x10^-3^ |
|  | **1500-2000** | 0 | 2.87 x10^-6^ | 7.25 x10^-6^ | 1.65 x10^-5^ | 5.91 x10^-4^ |
|  | **2000-2500** | 0 | 2.70 x10^-6^ | 7.25 x10^-6^ | 1.80 x10^-5^ | 5.27 x10^-4^ |
|  | **2500-3000** | 0 | 2.70 x10^-6^ | 6.24 x10^-6^ | 1.51 x10^-5^ | 1.08 x10^-4^ |
|  | **>3500** | 0 | 2.70 x10^-6^ | 6.24 x10^-6^ | 1.42 x10^-5^ | 9.11 x10^-4^ |
|  | **>3500** | 0 | 2.46 x10^-6^ | 5.93 x10^-6^ | 1.61 x10^-5^ | 1.87 x10^-3^ |

**S3 Fig: Cancer displays a distinct mutational pattern relative to normal based on the evolutionary age of genes.** For each human gene, the expected number of mutations is obtained based on the normal mutation pattern: frequency of normal mutations times the total number of cancer mutations (ICGC release 19) recorded in the data set. According to this, an Enrichment Ratio (ER) is calculated as the ratio of observed cancer mutations and the number of expected mutations in the gene. Then we define six different gene categories according to the level of enrichment. Then for each of these categories we produce an age distribution: Unexpected mutated genes are those genes that are never normally mutated but are mutated in cancer; Severely over-mutated genes are those that have more than 10 times more mutations in cancer than normal (ER>10); Moderately over-mutated genes are mutated in cancer 1.5 to 10 times more than normal (10>ER>1.5); Unaffected genes are genes with more or less the same number of mutations in cancer and normal (1.5>ER>0.67); Moderately under-mutated genes are mutated up to ten times less than normal (0.67>ER>0.1) and Severely under-mutated genes are those that are mutated more than 10 times less than normal, including seven genes that normally mutate but are never found mutated in cancer. Numbers in legend indicate the size of each gene set. Cross marks (X) on bars tips indicate the enrichment in that category is statistically significant according to a bootstrap test.

## Functional Enrichment of Human Genes as a Consequence of Evolutionary Age

The observed increase in mutational probability for young genes begs the question of whether this is the result of a mechanistic process by which ancient and highly conserved genes (and functions) are preserved over time, or whether the functions of the genes themselves contribute to the observed mutational skew, e.g. genes with functions that benefit from rapid evolution are young. This question prompted us to investigate gene functional enrichment with respect to age using DAVID [6–8] (S1 Table) to trace the evolution of diverse cellular and organismal functions. We grouped genes into age bins of widths between 38 million and 1000 million years, such that each group had between 1000 and 3000 genes (this was a limitation imposed by DAVID’s functional clustering tool). In this analysis it is important to note that the enriched functions represent the present situation and do not necessarily reflect the function of the ancestral genes. For instance, meiosis and oocyte development appear as a functional category for genes that are between 1.5 and 2 billion years old, prior to the emergence of sexual reproduction. Investigation of the genes driving this enrichment revealed that they are specifically involved in homologous recombination (HR), an important feature of sexual reproduction as well as DNA repair. The enrichment results support the idea that genes evolved first and the pathways in which they are employed evolved later. An example of this is that enriched genes involved in multicellular development are at least 100 million years older than the accepted evolutionary age of complex multicellularity[9].

**S1 Table: Evolution of biological functions as determined by gene function enrichment.**

See file S1_Table.xlsx

Our analysis yields a fascinating account of the evolution of successively more sophisticated cellular and multicellular mechanisms. As might be expected, the oldest gene functions were associated with the basic biochemistry of life – electron transport, nucleic acid metabolism, and sub-cellular localization. The youngest genes relate to more recently involved functions, such as the immune system, the sense of smell, keratinocyte development, pancreatic ribonucleases, and spermatogenesis. Among the most ancient features (2 to 2.5 billion years ago) are elements of signal transduction pathways that are often subject to targeted therapy in cancer, which appeared when the Ras-Rho axis rapidly differentiated into many small Ras-GTPases, accompanied by the evolution of RIG1, NOD, and MAP kinase signaling. Additional layers of regulation and feedback then evolved culminating with the appearance of Rho-GAP and Ras-GEF functions 1 to 1.25 billion years ago when simple multicellularity evolved. Complex multicellularity (800-900 million years ago) was preceded by the evolution of the genes involved in multicellular patterning and development such as WNT, TGFβ, FGF, Toll and NF-κβ signaling, cadherins, and metalloproteases that are involved in modifying the extracellular matrix such as ADAM. Immune system development and function dominates the period between 250 and 900 million years ago. In this category, genes involved in acute inflammatory responses evolved first, followed by secreted cytokines, wound healing, and the adaptive immune system. DNA damage recognition and repair systems evolved very early, but then underwent waves of refinement. The fundamental HR, base-excision repair (BER), nucleotide-excision repair (NER) and mismatch repair (MMR) pathways were enriched as long ago as 3-4 billion years, while younger DNA repair genes were generally involved in DNA damage recognition, checkpoint control, and cell cycle coordination.

**S2 Table: Details for functional enrichment network of recessive COSMIC cancer genes.** The network plot for the enrichment is shown in Fig 4. Each node in the network represents a group of functionally related genes as returned in DAVID (gene ontogeny, orthology, functional annotations, pathways, etc.). An additional level of clustering is represented by node colors defined in this table, revealing general functional associations of gene groups. Enrichment scores for each of these categories are shown.

See file S2_Table.xlsx

## The genomic distribution of SNV clustering differs between normal and cancer.

Distribution of SNV clustering by chromosome are shown as Circos plots in S3 Fig, S4 Fig and S5 Fig. Tracks from inside out are: blue, evolutionarily re-used breakpoint regions (EBR); green, amniote homologous synteny regions (mHSB); orange, hot spots of CM clusters in normal; and red, hot spots of CM clusters in cancer. Outside text track are symbols for COSMIC genes in their corresponding genomic locations. Dominant genes are in black fonts and recessive genes are in red font.

**S4 Fig: Circos plot showing distribution of SNV clustering by chromosome. Chromosomes 1 to 8**

**S5 Fig: Circos plot showing distribution of SNV clustering by chromosome. Chromosomes 9 to 16**

**S6 Fig: Circos plot showing distribution of SNV clustering by chromosome. Chromosomes 17 to 22 and X**

**Table C:** List of ICGC projects used in analysis for this paper.

| **ICGC Project Code** | **Samples Used in Analysis** | **Funding** | **References** |
| --- | --- | --- | --- |
| BOCA-FR | 20 | Institut National de la Santé et de la Recherche Medicale (Inserm) within the framework of the ICGC | Tirode et al. Genomic Landscape of Ewing Sarcoma Defines an Aggressive Subtype with Co-Association of STAG2 and TP53 Mutations. Cancer Discovery (2014), pp 1342-1353. doi: 10.1158/2159-8290.CD-14-0622 |
| EOPC-DE | 11 | Federal Ministry of Education and Research (BMBF) | Weischenfeldt et al. Integrative Genomic Analyses Reveal an Androgen-Driven Somatic Alteration Landscape in Early-Onset Prostate Cancer. Cancer Cell 23 (2013), pp 159–170. doi: 10.1016/j.ccr.2013.01.002 |
| MALY-DE | 26 | Federal Ministry of Education and Research (BMBF) | Alexandrov et al. Signatures of mutational processes in human cancer. Nature 500 (2013), pp 415–421. doi:10.1038/nature12477 |
| OV-AU | 115 | National Health and Medical Research Council (NHMRC) Queensland State Government University of Queensland Institute for Molecular Bioscience | Patch et al. Whole–genome characterization of chemoresistant ovarian cancer. Nature 521 (2015), pp 489–494. doi:10.1038/nature14410 |
| PACA-AU | 175 | National Health and Medical Research Council (NHMRC) Queensland State Government University of Queensland Institute for Molecular Bioscience  The Cancer Council NSW Garvan Institute of Medical Research Cancer Institute NSW | a) Waddell at al. Whole genomes redefine the mutational landscape of pancreatic cancer. Nature 518 (2015), pp 495–501. doi:10.1038/nature14169 b) Bailey et al. Genomic analyses identify molecular subtypes of pancreatic cancer. Nature 531 (2016) pp 47-52. doi:10.1038/nature16965. |
| PACA-IT/PAEN-IT | 31 | Italian Ministry of Education, University, and Research University of Verona | a) Bailey et al. Genomic analyses identify molecular subtypes of pancreatic cancer. Nature 531 (2016) pp 47-52. doi:10.1038/nature16965.  b) Scarpa et al (2016) under review |
| PAEN-AU | 43 | National Health and Medical Research Council (NHMRC) Queensland State Government University of Queensland Institute for Molecular Bioscience  The Cancer Council NSW Garvan Institute of Medical Research Cancer Institute NSW | Bailey et al. Genomic analyses identify molecular subtypes of pancreatic cancer. Nature 531 (2016) pp 47-52. doi:10.1038/nature16965. |
| PBCA-DE | 3 | Federal Ministry of Education and Research (BMBF) German Cancer Aid (DKH) | Jone et al, Dissecting the genomic complexity underlying medulloblastoma. Nature 488 (2012), pp 100–105. doi:10.1038/nature11284 |
| PRAD-CA | 123 | Ontario Institute for Cancer Research Prostate Cancer Canada | a) Boutros et al. Spatial genomic heterogeneity within localized, multifocal prostate cancer. Nature Genetics 47 (2015), pp 736–74. doi:10.1038/ng.3315 b) Cooper et al. Analysis of the genetic phylogeny of multifocal prostate cancer identifies multiple independent clonal expansions in neoplastic and morphologically normal prostate tissue. Nature Genetics 47 (2015), pp 367–372. doi:10.1038/ng.3221 |
| PRAD-UK | 62 | Cancer Research UK | Cooper et al. Analysis of the genetic phylogeny of multifocal prostate cancer identifies multiple independent clonal expansions in neoplastic and morphologically normal prostate tissue. Nature Genetics 47 (2015), pp 367–372. doi:10.1038/ng.3221 |
| SKCA-BR | 59 | Pio XII Foundation - Barretos Cancer Hospital René Rachou Research Center (FIOCRUZ) | In preparation. See <http://docs.icgc.org/portal/publication/> for information about publication restrictions. Data in this analysis published after receiving permission as per ICGC guidelines. |

## Supplemental References

1. Cunningham F, Amode MR, Barrell D, Beal K, Billis K, Brent S, et al. Ensembl 2015. Nucleic Acids Res. 2015;43: D662–D669. doi:10.1093/nar/gku1010

2. NCBI Resource Coordinators. Database resources of the National Center for Biotechnology Information. Nucleic Acids Res. 2015;43: D6-17. doi:10.1093/nar/gku1130

3. Penel S, Arigon A-M, Dufayard J-F, Sertier A-S, Daubin V, Duret L, et al. Databases of homologous gene families for comparative genomics. BMC Bioinformatics. 2009;10 Suppl 6: S3. doi:10.1186/1471-2105-10-S6-S3

4. Hedges SB, Dudley J, Kumar S. TimeTree: a public knowledge-base of divergence times among organisms. Bioinformatics. 2006;22: 2971–2972. doi:10.1093/bioinformatics/btl505

5. Huang D, Sherman BT, Tan Q, Collins JR, Alvord WG, Roayaei J, et al. The DAVID Gene Functional Classification Tool: a novel biological module-centric algorithm to functionally analyze large gene lists. Genome Biol. 2007;8: R183. doi:10.1186/gb-2007-8-9-r183

6. DAVID Bioinformatics Resources 6.7 (NIAID/NIH). Gene Functional Classification Tool [Internet]. Available: http://david.abcc.ncifcrf.gov/gene2gene.jsp

7. DAVID Bioinformatics Resources 6.7 (NIAID/NIH). Functional Annotation Tool [Internet]. Available: http://david.abcc.ncifcrf.gov/summary.jsp

8. Huang DW, Sherman BT, Tan Q, Kir J, Liu D, Bryant D, et al. DAVID Bioinformatics Resources: expanded annotation database and novel algorithms to better extract biology from large gene lists. Nucleic Acids Res. 2007;35: W169–W175. doi:10.1093/nar/gkm415

9. Narbonne GM. Evolutionary biology: When life got big. Nature. 2011;470: 339–340. doi:10.1038/470339a
