# Supplementary figures and images for: Ancient genes establish stress-induced mutation as a hallmark of cancer
Source: PLoS One. 2017 Apr 25;12(4):e0176258. doi: 10.1371/journal.pone.0176258 (PMC5404761; doi:10.1371/journal.pone.0176258)

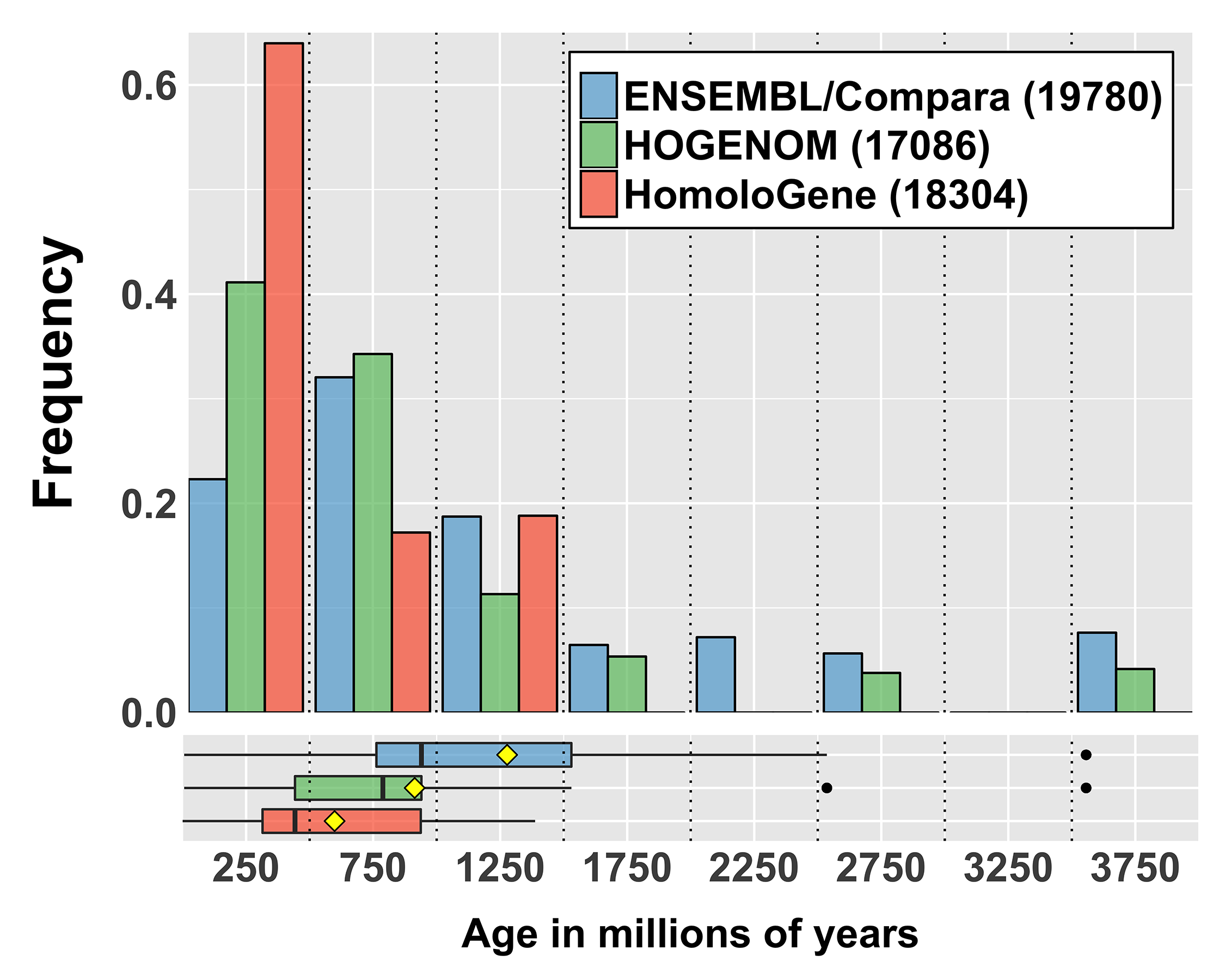

Supplement: S1 Fig — HomoloGene fails to reveal tree nodes corresponding to events of early evolution (older than 1500 MY), in turn giving a relative over-representation of resent events (less than 500 MY). The evolutionary time spanned by HomoloGene is later than the evolution of multicellularity. (TIFF) [file pone.0176258.s002.tiff]

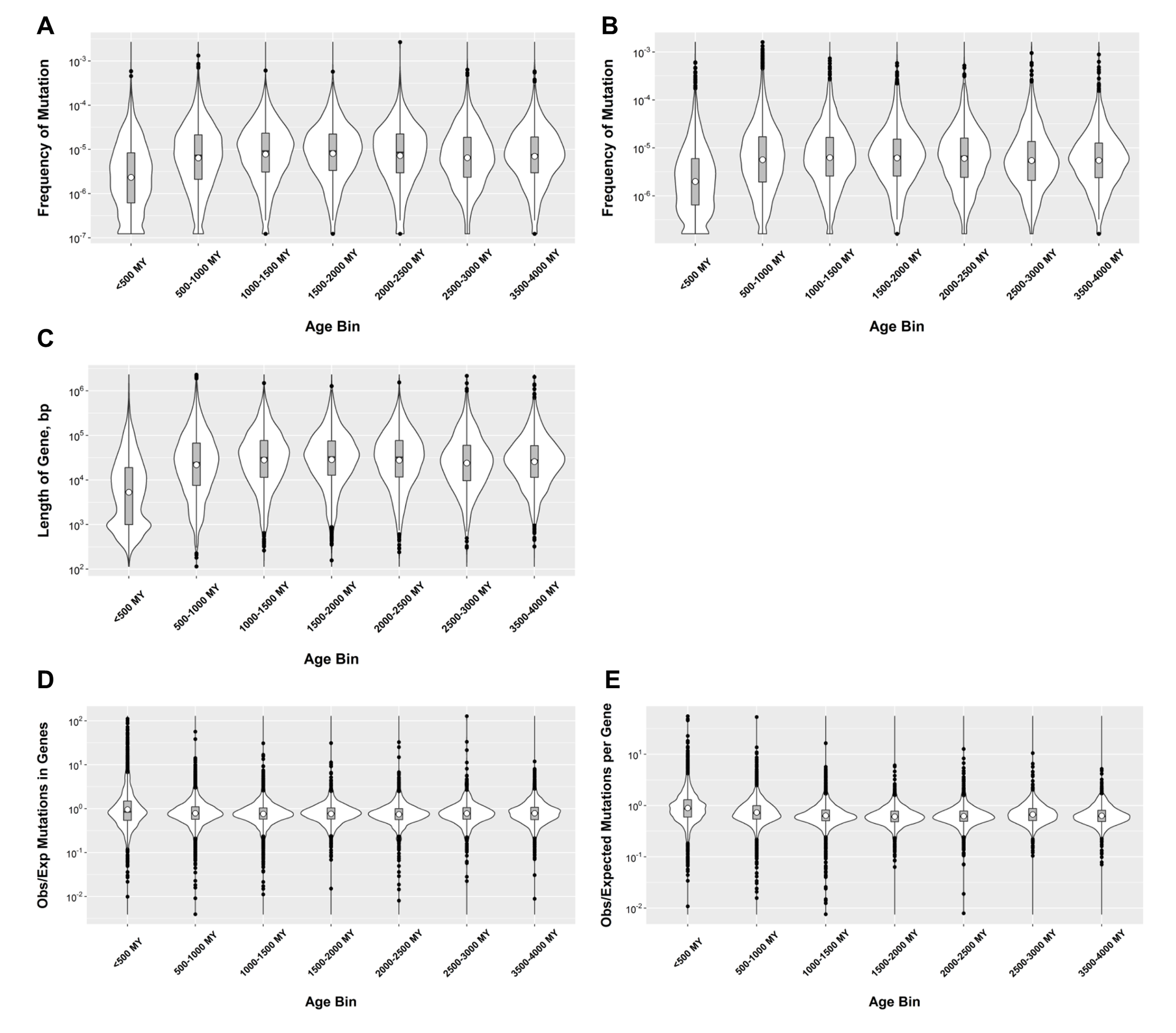

Supplement: S2 Fig — Frequency of Gene Mutation according to gene age. The distribution of values of mutation frequencies for each age group is estimated and shown as vertical violin and box plots. Horizontal lines are the median; circle is the mean and black dots are distribution outliers in each case. Vertical axis is in log scale. Corresponding plots are shown for both normal (A) and cancer data (B). In both cases it is evident that genes the first age bin (age < 500 MY) are typically mutated less frequently than the rest. (C) Distribution of gene lengths according to age group membership. Young genes are typically shorter than other genes. Frequency of gene mutation normalized by gene length for both normal (D) and cancer data (E) shows that young genes are more likely to be mutated. Groups were compared via ANOVA followed by Tukey’s Post-Hoc test to determining which relationships were driving the partitioning of variation. In normal (D), the <500 MY age bin is more frequently mutated compared to all other age bins (for all pair-wise comparisons, p<2.2x10^-16^). In cancer (E), the <500 MY age bin is more frequently mutated compared to all other age bins (for all pair-wise comparisons, p<2.2x10^-16^). Additionally, the 500–1000 MY age bin was more frequently mutated compared to 1000–1500 MY (p = 10−7), 1500–2000 MY (p = 3.2x10^-6^), and 2000–2500 MY (p = 3.6x10^-4^). (TIF) [file pone.0176258.s003.tif]

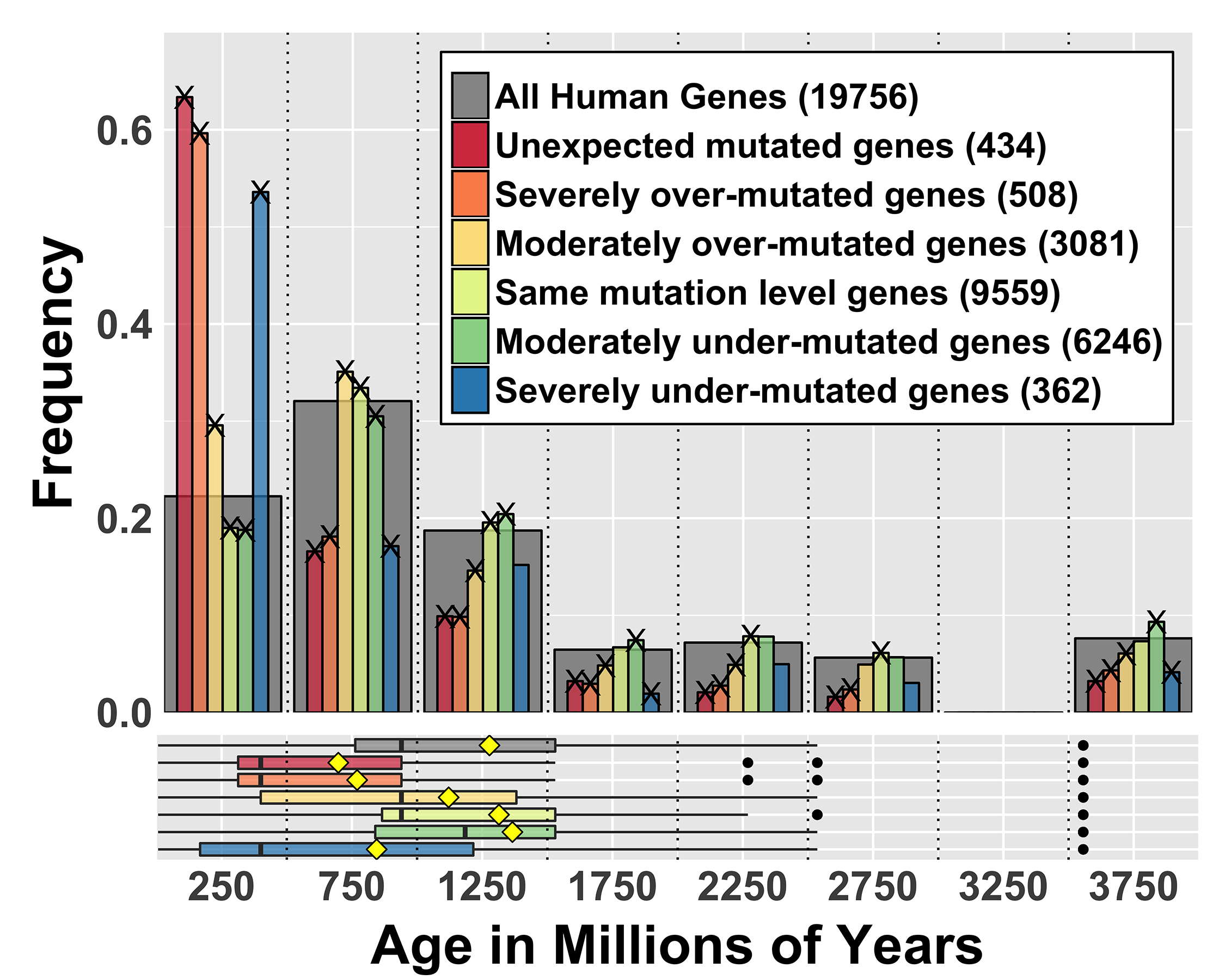

Supplement: S3 Fig — For each human gene, the expected number of mutations is obtained according to the normal mutation pattern: frequency of normal mutations times the total number of cancer mutations. The Enrichment Ratio (ER) is the ratio of observed cancer mutations and the number of expected mutations in the gene. We define six different gene categories according to the level of enrichment and produce age distributions. Unexpected mutated genes are those genes that are never normally mutated but are mutated in cancer; Severely over-mutated genes are those with over 10 times more mutations in cancer than normal (ER>10); Moderately over-mutated genes are mutated 1.5 to 10 times more in cancer than normal (10>ER>1.5); Unaffected genes have more or less the same number of mutations in cancer than normal (1.5>ER>0.67); Moderately under-mutated genes are mutated up to ten times less than normal (0.67>ER>0.1); and Severely under-mutated genes are mutated more over 10 times less than normal, including a few genes that normally mutate but are never found mutated in cancer. Numbers in legend are the sizes of each gene set. Cross marks (X) on bars tips indicate the enrichment in that category is statistically significant according to a bootstrap test. (TIFF) [file pone.0176258.s004.tiff]

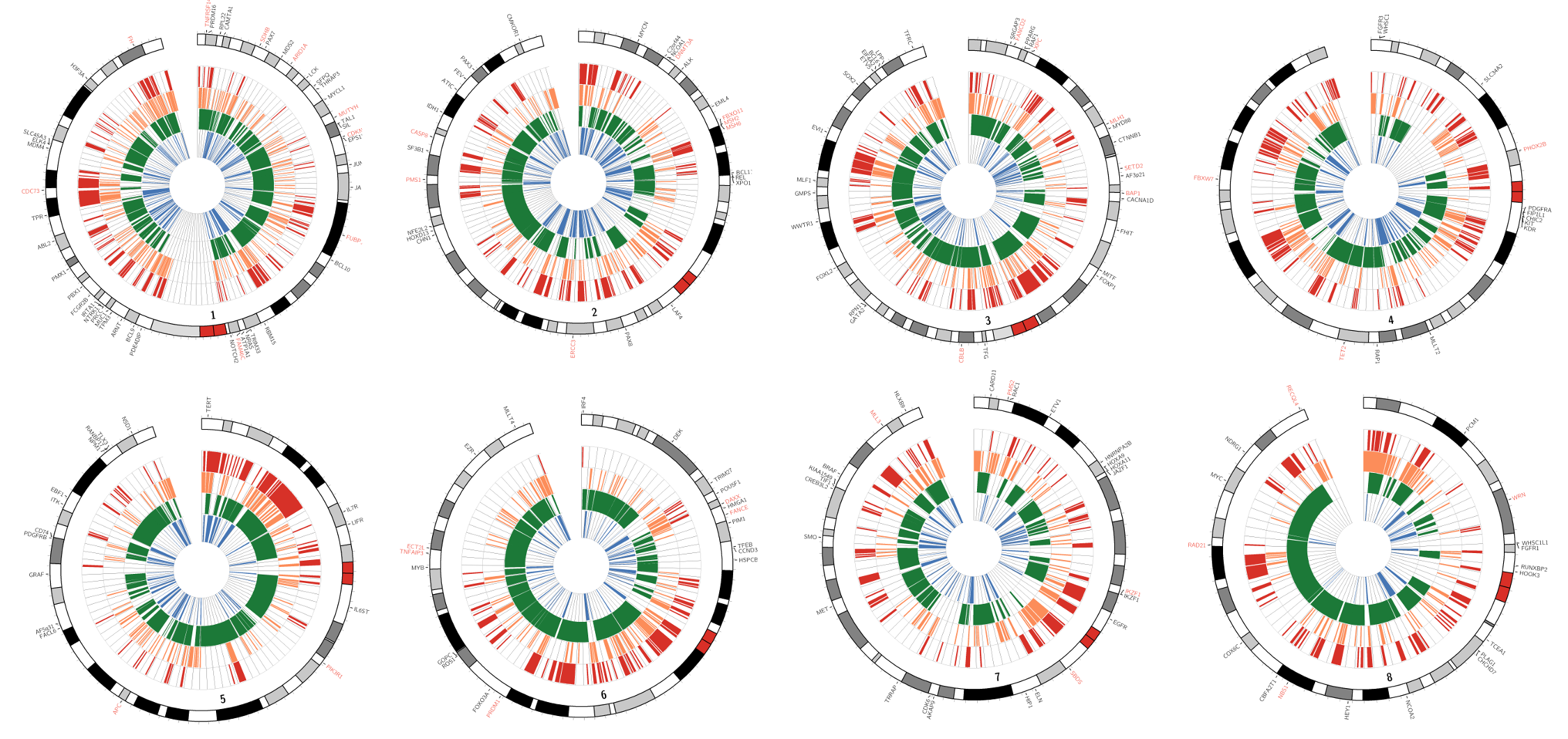

Supplement: S4 Fig — Chromosomes 1 to 8. (TIF) [file pone.0176258.s005.tif]

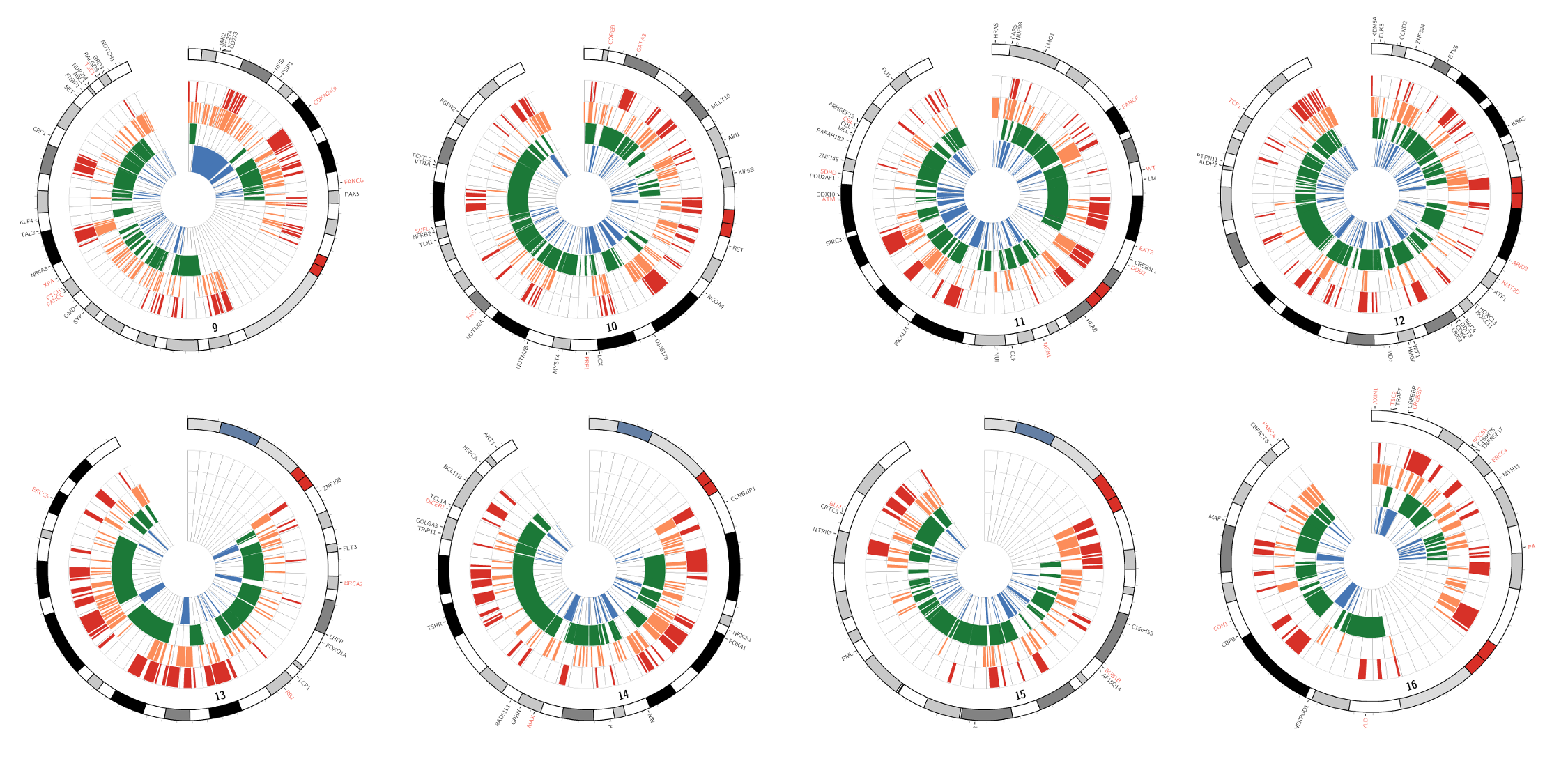

Supplement: S5 Fig — Chromosomes 9 to 16. (TIF) [file pone.0176258.s006.tif]

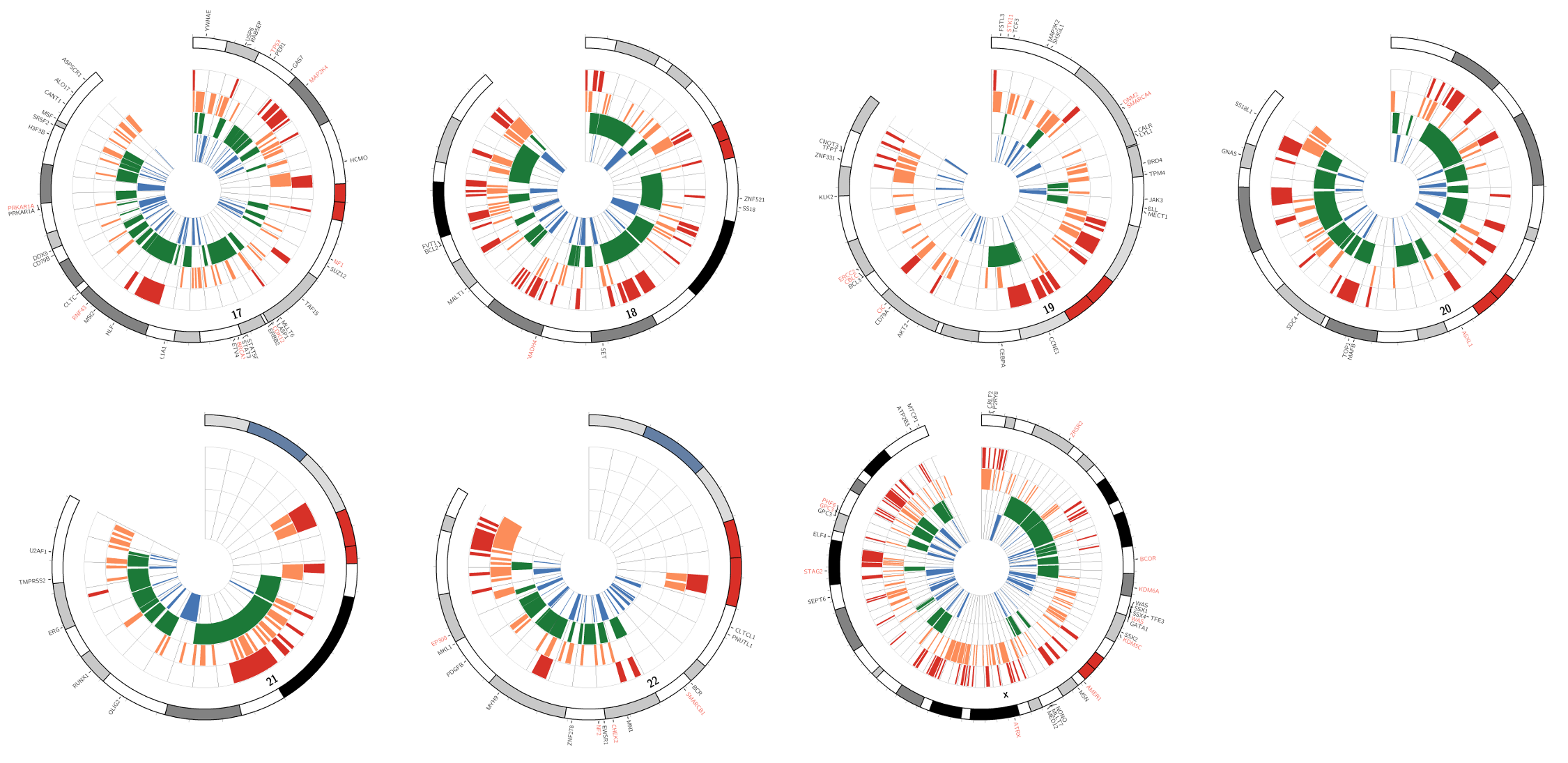

Supplement: S6 Fig — Chromosomes 17 to 22 and X. (TIF) [file pone.0176258.s007.tif]
